# Can consumer wearables support outpatient health monitoring for patients with post-acute infection syndromes? A systematic umbrella review of accuracy, validity, and clinical utility data

**DOI:** 10.1371/journal.pdig.0001124

**Published:** 2026-06-08

**Authors:** Deanna M. Kaplan, Nicole Kessler, Nicole S. Pozzo, Caroline Y. Doyle, Page Dickey, Nicholas A. Giordano, Asmita Lehther, Saad Maan, Jennifer S. Mascaro, Ariel McDowall, Shenita Peterson, Himani Sirsi, Roman Palitsky

**Affiliations:** 1 Department of Family and Preventive Medicine, Emory University School of Medicine, Atlanta, Georgia; 2 Department of Spiritual Health, Emory Healthcare, Atlanta, Georgia; 3 Department of Psychiatry and Behavioral Sciences, Rush University Medical Center, Chicago, Illinois, United States of America; 4 Nell Hodgson Woodruff School of Nursing, Emory University School of Medicine, Atlanta, Georgia; 5 Woodruff Health Sciences Library, Emory University, Atlanta, Georgia; 6 Department of Psychiatry, Emory University School of Medicine, Atlanta, Georgia; Peng Cheng Laboratory, CHINA

## Abstract

An estimated 65 million individuals around the world have experienced Post-Acute Sequelae of SARS-CoV-2 infection (PASC or Long COVID) and an additional 17–24 million are living with other post-acute infection syndromes. Current frontline treatment recommendations for these heterogenous, multisystemic conditions emphasize self-monitoring and non-progressive rehabilitation of activity and exertion. Consumer health wearables can potentially support symptom, activity, and exertion tracking; however, the strength of the scientific evidence supporting their use in this context is unclear. This pre-registered systematic umbrella review aimed to address this gap by synthesizing review-level evidence for the accuracy, validity, and clinical utility of consumer wearable–assessed biometrics relevant to PASC and related syndromes. Following PRISMA guidelines, 3,988 records were screened, with 42 reviews meeting inclusion criteria spanning more than 30 brands and over 150 distinct device series. This umbrella review characterized quality and risk of bias, synthesized and evaluated accuracy evidence for 17 biometrics, and evaluated clinical utility outcomes. Findings revealed highly variable review quality; substantial heterogeneity in device performance across biometrics, populations, and settings; and limited evidence for clinical utility. Two biometrics—heart rate measurement and atrial fibrillation detection—had comparably stronger support. Strengths and limitations to the current evidence base are identified, along with recommendations for informed patient use and the further research needed to responsibly support the integration of consumer wearables into outpatient care pathways. A living umbrella review repository is provided on the Open Science Framework so that findings can be updated as new, review-level evidence for emergent technologies becomes available.

## Author summary

Millions of people worldwide live with long-lasting symptoms after viral infections, including Long COVID. Many are encouraged to track their daily symptoms, activity, and physical limits to better understand their health and avoid setbacks. Consumer wearables—such as smartwatches and fitness trackers—offer a convenient way to do this, but it is not yet clear whether the science behind these devices is strong enough to support this recommendation. In this study, we reviewed and summarized review-level evidence on how accurate and clinically useful wearable-based measures are for health concerns relevant to Long COVID and other post-infection conditions. We examined thousands of articles and identified 42 reviews covering many brands, device types, and wearable features. Overall, we found inconsistent device accuracy across groups and settings, and limited evidence for clinical benefit. Two areas—heart rate measurement and atrial fibrillation detection—showed comparatively stronger support. Because technology evolves quickly, we created this project as a *living* review that can be updated as new evidence and new devices emerge. By outlining what is known, where the gaps are, and what kinds of research are most needed, our goal is to help researchers, clinicians, and patients make informed decisions about when and how wearables might be useful in outpatient care.

## Introduction

Consumer health wearables—commercially available health tracking devices such as smartwatches and other wearable sensors coupled to smartphones—have become broadly adopted in the United States. A 2024 study found that 44.5% of Americans reported using a wearable on a regular basis [1]. These devices are designed to passively and continuously collect physiological data, offering users a convenient, data-driven, and readily available means of engaging with indicators of their health. Biometrics tracked by consumer wearables with demonstrated links to health status include heart rate, heart rate variability, blood oxygen saturation, electrodermal activity, blood pressure, and indices of sleep quantity and quality, among others [2–4].

Consumer health wearables have been extensively applied for self-monitoring in healthy adult populations for purposes that include increasing physical activity [5], improving cardiovascular fitness [5,6], and enacting health behavior changes such as increasing daily movement or improving sleep quality [7,8]. Some consumer wearables have obtained United States (U.S.) Food and Drug Association (FDA) clearance for specific clinical assessment functions (e.g., arrhythmia detection or atrial fibrillation screening), but most are not approved in the U.S. for clinical monitoring or the management of medical conditions.

There is recognized potential for consumer wearables to play a more central role in health management, particularly for ongoing monitoring of chronic conditions outside of acute care settings [9–11]. The recent epidemic of Post-Acute Sequelae of SARS-CoV-2 infection (PASC), commonly known as Long COVID [12], brought the need for accessible and scalable self-monitoring technologies into increased public awareness. Symptoms of PASC are heterogenous and multisystemic, with similarities to other post-acute infection syndromes such as myalgic encephalomyelitis/chronic fatigue syndrome (ME/CFS) [13,14] and post-treatment Lyme disease syndrome [15]. The most frequently reported symptoms of PASC are either cardiopulmonary (e.g., shortness of breath, tachycardia, dizziness on standing) or secondary to cardiovascular symptoms (e.g., post-exertional malaise, fatigue, sleep disturbance) [16,17]. Although there are few targeted treatments for PASC or other post-acute infection syndromes, treatment guidelines largely emphasize patient self-monitoring and self-paced, non-progressive rehabilitation (e.g., “careful avoidance of situations that exacerbate symptoms” [16], or “symptom-guided pacing rehabilitation” [18]. For the estimated 65 million individuals who have experienced PASC and the additional 17–24 million who are living with other post-acute infection syndromes [19], this is an ambiguous recommendation that provides little concrete guidance for implementation.

Individuals who seek to take up this guidance may naturally turn to wearable technologies, and indeed, consumer health wearable sensors have been proposed as a scalable and already-broadly accessible support for patients’ self-monitoring needs [20,21]. Consumer health wearables are low-cost compared to prescribed, medical-grade devices. For example, the popular Fitbit Charge 6 (which measures heart rate, skin temperature, sleep quality and quantity electrodermal activity, physical activity, and also notifies users of “signs of atrial fibrillation”) retailed for $159 USD in April 2026 [22]. Research has identified significant differences in wearable-assessed resting heart rate and activity patterns between individuals who do and do not carry a diagnosis of PASC [23], suggesting that cardiac activity trackers may be useful for PASC self-monitoring. A recent survey study of PASC and ME/CFS patients who use a wearable tracker found that the vast majority perceived it to be helpful: 94% reported improved understanding of their daily energy budget and 85% felt increased control over their symptoms [21]. If wearables can accurately track biometrics relevant to patients’ symptom self-monitoring and management, they could provide an affordable alternative to medically prescribed tracking technologies. Used appropriately, wearables could then inform self-management as well as clinical decision-making in collaboration with a healthcare provider.

Despite growing enthusiasm for the clinical potential of wearables—and the nearly 20,000 scientific publications about their applications to healthcare, according to one bibliometric analysis [24]—the scientific evidence supporting their clinical use in outpatient health monitoring remains unclear. Questions persist regarding the reliability of wearable-derived metrics across diverse populations, the alignment of these metrics with gold-standard clinical measurement methods, and the extent to which wearable data can meaningfully inform diagnosis, treatment, or symptom management. In response to these questions, this systematic umbrella review aimed to (1) characterize the review-level evidence for the accuracy, reliability, and clinical utility of consumer wearables for monitoring biometrics with relevance to PASC and other post-acute infection syndromes, and (2) identify patient characteristics or contexts of wearable use associated with reduced wearable-assessed data quality. By consolidating findings across prior systematic reviews and meta-analyses, this work aims to clarify the state of the science and identify areas in which further research is needed to inform the responsible implementation of wearables to support patient self-monitoring in PASC and post-infection syndromes with similar clusters of symptoms.

## Materials and methods

A systematic umbrella review (i.e., a review of reviews) was conducted in accordance with PRISMA guidelines. The review was pre-registered in PROSPERO: [https://osf.io/c79du].

### Search methods

In February 2023, a search was conducted in PubMed, Embase *(Elsevier)*, Global Health (CABI), IEEE Xplore*,* and Web of Science (Clarivate) which includes the following indexes: Science Citation Index Expanded (SCIE), Social Sciences Citation Index (SSCI), Arts & Humanities Citation Index (AHCI), Emerging Sources Citation Index (ESCI), Conference Proceedings Citation Index (CPCI), Book Citation Index (BKCI), Current Chemical Reactions (CCR), Index Chemicus (IC)). The search used a combination of keywords for the concepts of ‘consumer wearable’ and ‘reviews’; the full list of 31 search terms and Boolean operators is provided in S1 Appendix, along with the search strategy for PubMed and other databases. The concept of “consumer wearable” included all consumer devices designed for continuous wear on the body, such as wearable activity trackers, wristwatches, and smart jewelry such as rings. The search did not include additional limits. The search result records were imported into EndNote 20 for data management.

### Article screening and assessment for eligibility

Inclusion and exclusion criteria are provided in full on the Open Science Framework (osf.io/c79du). Given that PASC only emerged as a recognized clinical diagnosis in 2020 and post-acute viral syndromes are broadly recognized to be under-researched, we intentionally applied broad search and inclusion criteria for this review. Inclusion criteria were selected for relevance to conditions and domains that correspond with post-infection syndromes, as well as insights from general biometric monitoring research that bear on self-monitoring for these conditions. Inclusion criteria were: (1) reviews (including systematic and non-systematic reviews and meta-analyses of primary empirical research); (2) evaluating accuracy, validity, feasibility, or clinical utility; (3) of commercially available consumer health wearables designed for continuous wear over a full day’s activities; (4) with sensors capable of acquiring data with relevance to PASC and post-infection syndromes (e.g., heart rate), described in detail on the OSF supplement; (5) of adults 18 years of age or older.

Article screening was conducted in Covidence cloud-based systematic review software. For all records yielded by the literature search, titles and abstracts were independently screened for inclusion by pairs of trained research assistants. Disagreements were resolved by discussion, initially in team meetings and then independently by each pair of screeners, reaching 100% consensus. Records that met inclusion based on abstract screening subsequently underwent full text review by pairs of trained research assistants, with disagreements again resolved by discussion, initially in team meetings and then independently, reaching 100% consensus.

### Data extraction

An extraction template corresponding to the aims of this research was developed by the investigators, piloted in a randomly selected sample of k = 5 included reviews, and then refined before being applied to all included reviews. During the pilot, considerable variation in the detail with which reviews reported results and interpretive conclusions was observed. To account for this heterogeneity, the final version of the extraction template included four levels of reported information: results reported at the level of individual devices (e.g., Fitbit Charge 2), at the level of device brands (e.g., Fitbit), at the level of types of sensors (e.g., accelerometer), and at the level of categories of devices (e.g., wristwatches vs. chest straps). Extracted variables are summarized below. The full extraction template used for this project is made publicly available on the Open Science Framework at osf.io/c79du.

Extraction was initially conducted using Covidence, and then transitioned to Microsoft Excel due to the large size of the extraction template used for this review. Double extraction was employed, such that two team members independently extracted each variable from each article. Disagreements were resolved by discussion, initially during team meetings and then independently, reaching 100% consensus. For reviews that included devices intended for use in research and clinic settings as well as commercially available consumer devices, per the review criteria, information was only extracted for the commercially available devices unless the clinical-grade device was specifically used for benchmarking (in which case comparisons with clinical grade devices were extracted when available). When it was unclear whether a device was intended for commercial availability, determination about inclusion in this study was made based on whether it was possible to purchase the device as a private consumer, without an academic or medical affiliation and without specialized training for use. Products exclusively targeting research or clinical markets (e.g., the widely published-on but recently discontinued ActiGraph GT9X) were excluded from this review as non-consumer products. Data from devices marketed to both research and public consumers were included in the review.

Information was extracted about the type of review (e.g., narrative review, systematic review, meta-analysis), review aim, number of primary studies included, and conflicts of interests disclosed by the authors. Information about any risk of bias assessment completed by each review, including the assessment tool(s) and the results of that assessment, was extracted. Missing data (e.g., variables in the extraction template that were not included in a given review) were extracted as “not included”.

#### Populations, contexts, and devices reviewed.

Demographic and clinical or morbidity characteristics of targeted and included populations were extracted, as well as the characteristics of contexts in which the evaluated devices were used. These included: type of setting (e.g., naturalistic vs. controlled), presence of any medical oversight, and any co-occurring medical interventions such as medications.

#### Biometrics.

Data were extracted for the following biometrics: heart rate (HR), heart rate variability (HRV), blood pressure, atrial fibrillation detection, other arrhythmia detection, EKG, oxygen saturation (O2sat), V02Max, skin temperature, electrodermal activity (EDA; also sometimes called galvanic skin response or GSR), steps taken, flights of stairs climbed, energy expenditure (EE, estimated amount of energy burned by the body), total sleep time (TST), sleep efficiency (SE), sleep onset latency (SOL), and wake after sleep onset (WASO).

**Validity-related variables for biometrics.** For all biometric variables, indices of validity extracted included: accuracy (e.g., reported data about sensitivity, specificity, Mean Absolute Percentage Error (MAPE)), reliability (e.g., agreement of a device with itself over repeated assessments in the same or different contexts), and the comparator used for accuracy benchmarking (e.g., another wearable, a clinical device such as 12-Lead ECG). We additionally extracted determinants of accuracy reported by the review, spanning (a) demographic characteristics, (b) clinical or health morbidity characteristics, and (c) setting and context of use.

Reviews evaluating validity-related variables did so with varying degrees of specificity, including (1) appraisal of devices within a given category of devices (e.g., wristwatches vs. chest straps or patches), (2) appraisals of specific biometrics (e.g., heart rate), and (3) appraisals of specific individual devices (e.g., Fitbit Charge 2). Given the importance of distinctions between these levels of analyses, assessments were extracted for each of these levels of analysis. Differences were also observed between reviews in how terms such as “reliability” were used; for example, accuracy of a wearable device as benchmarked against laboratory equipment was sometimes interpreted by a review as “accuracy” and sometimes as “reliability.” Results of this umbrella review are reported with fidelity to the original language and interpretations of included reviews.

#### Clinical utility of devices.

Information about the reported clinical utility of devices for assessment, diagnosis, symptom monitoring, or symptom management are also reported. Guided by a multi-dimensional operationalization of clinical utility introduced by Smart, 2006 [25], the following variables were extracted: evidence of effectiveness for achieving intended clinical change; evidence of suitability for diagnostics; utility within clinical settings; patient acceptability; provider acceptability; accessibility; adherence; and tracking of adverse events and adverse effects.

### Quality assessment and corrected cover area matrix

Quality of included reviews was evaluated using the AMSTAR 2.0 framework [26]. Each study was independently assessed by two authors and disagreements were resolved through discussion, initially in team meetings and then independently, with 100% resolution. As recommended by Hennessy & Johnson (2020) [27], a corrected covered area (CCA) matrix was created from all reviews that reported a complete list of included studies (k = 20), to identify the degree of overlap, and thus potentially heightened influence, in the primary studies among the reviews that comprise this umbrella review.

## Results

Six thousand eight hundred and thirty-six (6,836) records were imported into the web-based application Covidence. After de-duplication, 3988 records were screened for inclusion.

**Fig 1:**
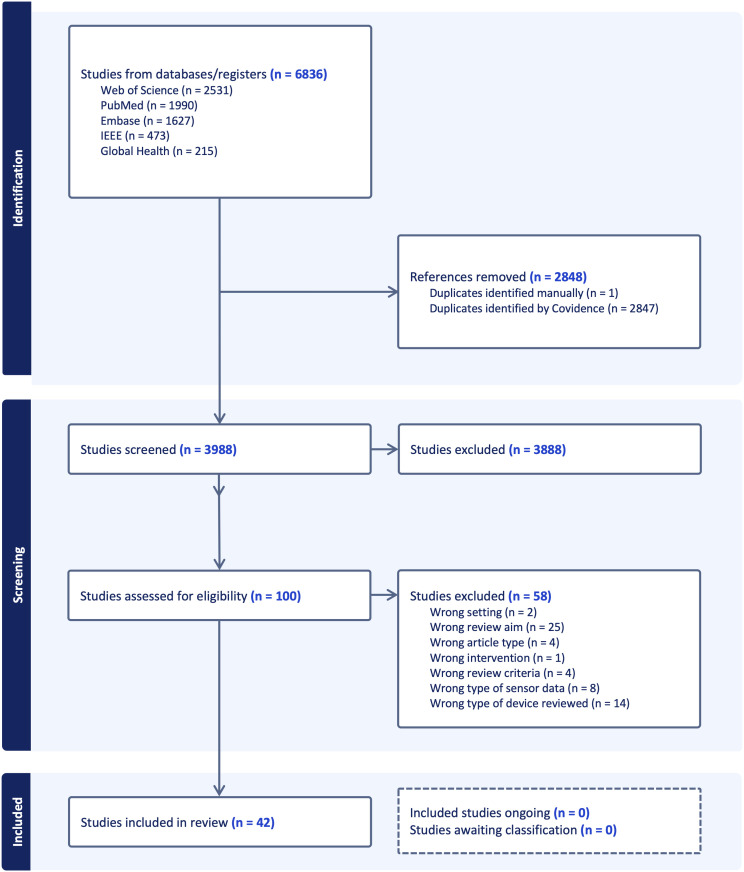
PRISMA Diagram. This PRISMA flow diagram is distributed under the terms of the Creative Commons Attribution (CC BY 4.0) license, which permits unrestricted use, distribution, and reproduction in any medium, provided the original work is properly cited. For the original published work, please see Page MJ, McKenzie JE, Bossuyt PM, Boutron I, Hoffmann TC, Mulrow CD, et al. The PRISMA 2020 statement: an updated guideline for reporting systematic reviews. BMJ 2021;372:n71. doi: 10.1136/bmj.n71.

The PRISMA diagram resulting from this work is provided as Fig 1.

Of the 3,988 reviews initially screened, 42 met inclusion criteria. These reviews included more than 30 brands and over 150 distinct device series. S2 Appendix lists all included reviews, the number of primary studies they included, as well as the devices covered by each review.

The CCA among included reviews was 3.1%. Reviews with under 5% CCA have only slight overlap and are not considered to be problematic for interpretation of results [28]. Of all 397 primary studies included across all reviews, 34% (k = 136) were included in more than 1 review. Five studies were identified that had exceptional influence (i.e., included in >4 reviews). In descending order, one study was included in 7 reviews [29] two studies were included in 6 reviews [30,31]; and two studies were included in 5 reviews [32,33]. The CCA matrix is provided on the Open Science Framework at osf.io/c79du.

### Characteristics of included reviews

#### Review aims.

Aims most frequently represented among study reviews were accuracy, validity, or reliability (k = 35), followed by clinical utility (k = 12), usability and accessibility (k = 8), and outcome/health improvement (k = 6).

#### Conflicts of interest.

Of the 42 articles in this review, 31 reported no conflicts of interest (COI) and four reviews did not contain a COI statement. Of the seven articles with a reported COI, only two reported a competing interest that included a direct connection to companies making the reviewed wearables, which were Garmin [34] and Apple [35]. In three additional reviews authors reported ties to medical technology and biotechnology companies [36–38].

#### Quality assessment.

Ratings are presented for all 16 AMSTAR 2.0 categories (or 13 if no meta-analysis was conducted) as a heat map in S3 Appendix, as the AMSTAR 2.0 is not intended to provide a total score. Quality was highly variable: among all reviews, the number of criteria to score “yes” or “partial yes” (indicating higher quality), out of a total possible 16 (or 13), ranged from 0-13 (*M* = 6.14, *SD* = 3.14). On average, the included reviews met approximately 44% of applicable AMSTAR 2.0 criteria as “yes” or “partial yes.” Categories most frequently demonstrating high quality (ratings of “yes”) were study selection in duplicate (AMSTAR 2.0 item 5), satisfactory explanation for heterogeneity observed in results (AMSTAR 2.0 item 14), and sources of conflict of interest (AMSTAR 2.0 item 16). Categories that most frequently did not merit “yes” were: list of excluded studies and justification for exclusion (AMSTAR 2.0 item 7) and satisfactory technique for assessing the risk of bias (RoB) in individual studies (AMSTAR 2.0 item 9).

#### Risk of bias (RoB).

RoB was completed by 16 included reviews. Of these, 7 concluded a low risk of bias, 2 concluded a medium risk of bias, 3 concluded a high risk of bias, and 4 reviews described a risk of bias assessment but did not draw clear conclusions about the risk of bias in their review. Reviews reporting risk of bias and their resultant conclusions are reported in S4 Appendix.

### Populations, contexts, and devices reviewed

Population characteristics are reported for both targeted populations (the populations *a priori* targeted by review authors) and included populations (the populations actually reported on in the review, for which data were available) (Table 1).‌‌

**Table 1:** Characteristics of participants.

Population Characteristics	Targeted Population	Included Population
	(k reviews)	(k reviews)
Any racial characteristic	1	3
Any ethnicity characteristic	0	2
Gender	1	29
Biological sex	0	0
Sexual orientation	0	0
Age (all)	14	33
Adults (18–65 years)	10	32
Older adults (65 + years)	9	25
Socioeconomic status	0	6
Geography (rural, urban, suburban)	0	3
Educational attainment	0	2
Veteran/military status	0	0
Other: occupational groups (all)	2	0
Athletes	1	0
Firefighters, roofers, office workers, construction workers	1	0
Clinical/morbidity characteristics (all)	5	21
Cardiovascular symptoms or events	5	21
Respiratory symptoms or diagnoses	2	12
Chronic pain	2	8
Body mass index (BMI)	2	7
Cancer	1	3
Medically healthy	1	20
Neurological events and conditions	0	8
Metabolic conditions	0	6
Sleep disturbances	0	5
Mental health symptoms or diagnoses	0	5
Orthopedic conditions	0	5
Other non-chronic characteristic (pregnancy, perimenopause, unspecified lower back pain)	0	6
Other chronic illnesses (fatigue, sickle cell, ME/CFS, POTS, MCAS, MS)	1	6

#### Settings and contexts of use.

The settings in which devices were evaluated (controlled lab or clinic settings, naturalistic or field settings, or both) were specified by 14 (33.3%) of the 42 included reviews. Of these, most (k = 12) reviews reported on both laboratory and naturalistic settings, with two reviews [39,40] reporting on controlled lab or clinical settings only. Medical supervision of device use was reported within four reviews. Of the 14 reviews specifying settings of use, only nine reported settings used in primary studies, such that the proportion of studies across settings could be computed (Table 2).‌‌ Averaged across these nine reviews, naturalistic or field settings accounted for 20% of studies reviewed, controlled lab or clinic settings accounted for 30.6% of studies reviewed, and mixed settings accounted for 45.9% of studies.

**Table 2:** Reported settings of wearable use among reviews.

Review	Naturalistic/Field k(%)	Laboratory/Clinic N (%)	Mixture of Both Setting Types N (%)	Setting Not Reported N (%)	Total k
Feehan et al., 2018	0 (0%)	0 (0%)	67 (100%)	0 (0%)	67
Irwin et al., 2022	2 (25%)	1 (12.5%)	1 (12.5%)	4 (50%)	8
Patel et al., 2021	0 (0%)	14 (100%)	0 (0%)	0 (0%)	14
Straiton et al., 2018	4 (57.1%)	2 (28.6%)	1 (14.3%)	0 (0%)	7
Reeder et al., 2016	6 (35.3%)	10 (58.8%)	1 (5.9%)	0 (0%)	17
Alam et al., 2022	2 (33.3%)	1 (16.7%)	1 (16.7%)	2 (33.3%)	6
Alharbi et al., 2019	12 (60%)	6 (30%)	2 (10%)	0 (0%)	20
McCullagh et al., 2016	4 (16.7%)	16 (66.7%)	4 (16.7%)	0 (0%)	24
Wright et al., 2017	4 (57.1%)	2 (28.6%)	1 (14.3%)	0 (0%)	7
All Studies N (%)	34 (20%)	52 (30.6%)	78 (45.9%)	6 (3.53%)	170

*Note.* This table only includes reviews that reported sufficient detail about settings of use to enable extraction of that information. Headers in the first column represent each review that contained such information, with information pertaining to each review provided on its row. Headers across the first row represent settings of use, which include: Laboratory/Clinic (studies conducted in closed settings controlled by researchers, such as a medical clinic or research laboratory), Naturalistic/Field (non-controlled settings of use, such as in participants’ homes, workplaces, or across a range of settings of daily life), a mixture of both of these setting types, or not reported. Alongside the number of studies in each category, “k%” refers to the percentage of studies from a given review conducted in the setting of use.

#### Evaluations of population and setting characteristic-based differences in device accuracy.

**Fig 2:**
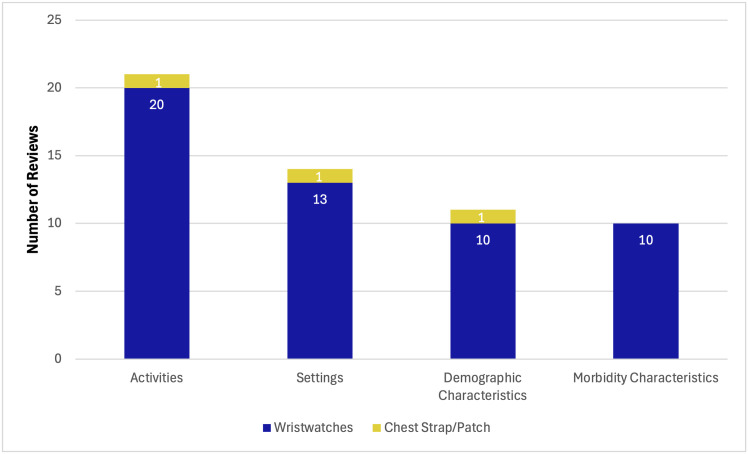
Number of studies reporting on factors that influence the accuracy of wearable devices (k = 29 reviews). Activities = differences in accuracy based on participants’ activity (e.g., what the participants were doing while wearing the device, such as sleeping compared to exercising vigorously). Settings = where the device was worn on the participants’ body. Demographic Characteristics = participant characteristics such as race, ethnicity, gender, or age. Morbidity Characteristics = participant health status, symptoms, or diagnoses. These categories are not mutually exclusive, as many reviews addressed more than one factor.

Sources of heterogeneity in accuracy such as characteristics of the user (e.g., demographics, user health status) or characteristics of use (e.g., where on the body the device was worn, activities performed during wear) were evaluated by k = 29 reviews (Fig 2). Detailed information about how these factors were reported to impact accuracy is organized by biometric and provided in the sections below.

### Validity-related variables (accuracy, reliability) for wearable-assessed biometrics

**Fig 3:**
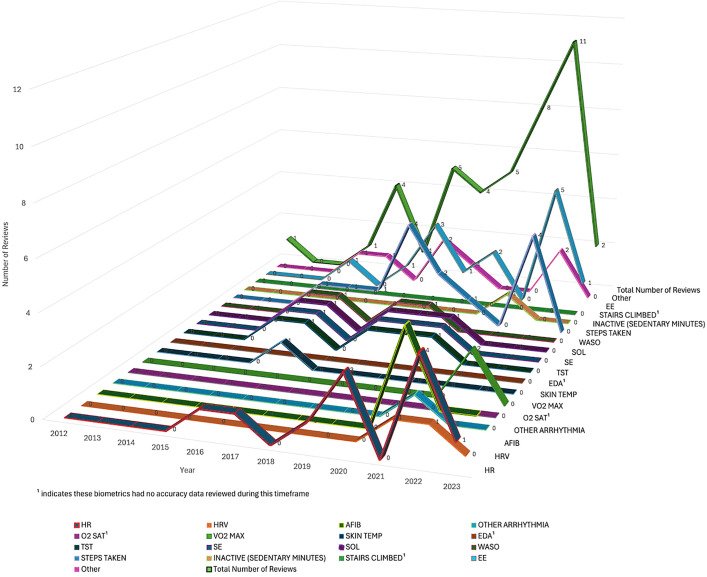
Availability of review-level evidence for biometrics over time. HR = heart rate, O2SAT=oxygen saturation, TST = total sleep time, HRV = heart rate variability, VO2MAX=estimate of maximum oxygen used during intense exercise, SE = sleep efficiency, AFIB = atrial fibrillation detection, SOL = sleep onset latency, EDA = electrodermal activity, WASO = wake after sleep onset, EE = energy expenditure.

**Fig 4:**
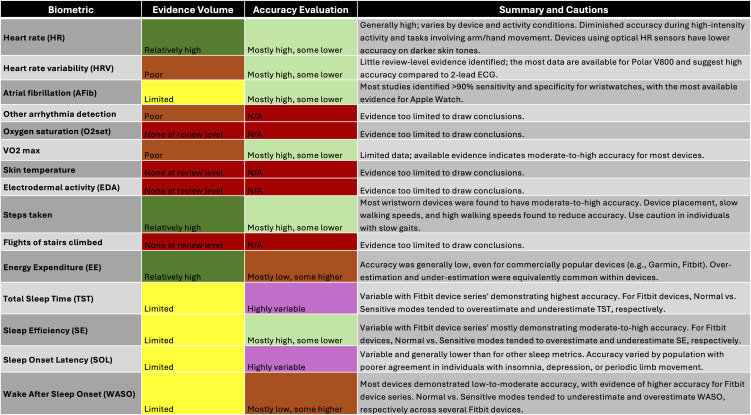
Heat map summarizing volume of evidence and overall accuracy evaluations for biometrics reviewed. For Evidence Volume, “relatively high” = 11 + reviews, “moderate” = 7–10 reviews, “limited” = 3–7 reviews, “poor” = 1–3 reviews, “none at review level” = 0 reviews. Accuracy Evaluation and Summary and Cautions columns provide a highly abbreviated summary of the detailed information for each biometric at the device-level and review-level that can be found in S5-S13 Appendices.

Review-level data were identified at the device level for most of our targeted biometrics, described below. The years from which review-level data were available are visualized in Fig 3; review-level evaluations became available beginning in 2016 for most variables, with most reviews represented in this umbrella review published in 2021 or 2022. Fig 4 provides a heat map illustrating the volume of evidence, accuracy evaluation, and overall summary for each biometric reviewed.

### Heart rate (HR)

15 reviews evaluated the accuracy of HR assessments for specific devices, spanning 42 distinct devices (see S5 Appendix for detailed reporting at the device level). Accuracy of heart rate data was benchmarked against clinical grade devices (k = 13) and against other consumer wearable devices (k = 7). The observed accuracy of individual devices was found to be inconsistent across reviews. Evaluations of accuracy varied across different benchmarking devices, settings of use, and evaluative methods used among the reviews. The most consistently high ratings across reviews (based on k = 7 reviews) were observed for Apple Watch, based on benchmarking against clinical grade (e.g., electrocardiogram: ECG) and consumer wearable devices.

**Determinants of accuracy.** Differences in accuracy based on population were evaluated by k = 5 reviews. Two reviews [41,42] reported accuracy differences between healthy adults and populations who had cardiac conditions, such as atrial fibrillation (AF). Within this population, Apple Watch 4 HR was evaluated as *more* accurate for patients with AF than for patients without AF. Conversely, Fitbit Blaze was found to underestimate HR in participants with cardiac conditions. One review [42] noted accuracy problems among individuals with cystic fibrosis: the Fitbit Charge HR was found to underestimate HR among adults with cystic fibrosis during exercise. One review [43] found greater accuracy for overweight populations compared to normal-weight participants. Two reviews [44,45] reported that HR measurement was less accurate in individuals with darker skin color, but noted that wearables using red spectrum light may be more accurate for darker skin tones due to lower red-spectrum absorption in melanated skin. Emerging evidence that individual differences in hair follicle density and sweat can also influence measurements was additionally noted, although the direction or and magnitude of this effect was not discussed [45].

Accuracy differences between settings of use were evaluated by k = 4 reviews, comprising different types of activities: cycling, conventional walking, Nordic walking, and high intensity activities. Lower accuracy during cycling was observed by multiple reviews [41,42,46]. Among Fitbit devices, lower accuracy was observed during high-intensity physical activity [42,47].

**Reliability.** Only k = 2 reviews addressed reliability of HR data. One review [48] reported the Fitbit Charge 2 to demonstrate reliability for HR assessments at low to moderate exercise levels. The second [34] noted that Apple Watch demonstrated high inter-device reliability during treadmill bouts, with reliability increasing and standard typical error decreasing as pace increased. In addition, Fitbit Charge 2 was found to demonstrate high reliability during treadmill bouts, but poor reliability during hand movement tasks (e.g., dusting) and Garmin Vivosmart HR+ demonstrated high reliability during all tasks assessed.

### Heart rate variability (HRV)

Two reviews evaluated accuracy of HRV estimates derived from consumer health wearables. One [49] evaluated Polar H10 and Polar V800 devices as having high accuracy, based on benchmarking against a 2-lead ECG. Another [41] evaluated HRV estimation models used by Apple Watch (device series not specified) in comparison with other estimation approaches. They reported that the device missed approximately 10% of beats, with a greater number of missing beats at the beginning of recording intervals, and concluded that Standard Deviation of Normal-to-Normal Intervals (SDNN) was acceptable for estimating HRV.

### Atrial fibrillation

Six reviews evaluated the accuracy of atrial fibrillation detection by consumer health wearables, spanning 16 distinct devices (see S6 Appendix for detailed reporting at the device level). Atrial fibrillation detection benchmarking was reported against clinical grade devices (e.g., 12-lead ECG) in nearly all reviews, with accuracy reported in terms of sensitivity and specificity. Nearly all devices covered were found to have high or moderate-to-high accuracy, with the exception of Fitbit Charge HR, which was evaluated as having low accuracy for atrial fibrillation detection [50]. Accuracy data from more than one review was available for two device brands. Samsung (inclusive of multiple device series) received high accuracy ratings across k = 5 reviews, with sensitivity reported as high as 97% [51] and specificity as high as 98% [52]. Apple Watch (inclusive of multiple device series) received mixed evaluations, with one review suggesting high accuracy with sensitivity of up to 97.92% and specificity of up to 99.61% [53], and another review reporting sensitivity as low as 41% [41].

### Physical activity

**Steps taken**. Sixteen reviews reported accuracy for wearable-assessed step count spanning a total of 42 devices (see S7 Appendix for detailed reporting at the device level). Reviews reported accuracy as assessed against a considerable range of comparators (e.g., ActiGraph, manual and observational step counts). Estimations of accuracy also varied, not only between devices but also between different reviews for the same devices. For example, one review reported strong agreement between the Fitbit Zip and ActiGraph counted steps [54], while two other reviews [55,56] noted very low agreement between the same device and direct observation.

***Determinants of accuracy.*** Four reviews evaluated differences in accuracy for specific populations, and found that accuracy varied based on gait speed, age, and overweight status. Gait speeds below 1 meter per second (m/s) resulted in overestimation across devices according to one review [57], but another review [47] observed the opposite inaccuracy at low gait speeds for Fitbit One specifically, which was found to record zero steps for participants walking at 0.3-0.5 m/s. Similarly, low accuracy for individuals with low gait speeds (e.g., walking trials of 0.3-0.9 m/s) was identified by another review [58] for wrist-worn and waist-worn wearable physical activity monitors. One review [43] evaluated differences in accuracy based on population characteristics, and reported diminishments in accuracy for older adults and overweight individuals.

Six reviews evaluated differences in accuracy based on activity type and data acquisition settings. The settings and uses evaluated by these reviews included walking up stairs, walking on an incline, sedentary behavior, household activities (e.g., laundry, making bed), laboratory settings, free-living (non-laboratory) environments, walking on a treadmill, and normal body movement vs. constrained body motion. Several accuracy discrepancies were identified. At the device level, Garmin activity trackers were found to underestimate steps when walking on flat ground and upstairs, but to overestimate steps when walking downstairs [59]; another review reported a difference in step count for the Garmin vivosmart HR in indoor vs outdoor locations [55]. Fitbits were found to overestimate steps in a non-laboratory environment, but the extent of overestimation depended on the criterion measure [60]. Appraising devices more broadly, one review [56] reported that wristwatch wearables overall had reduced accuracy for steps taken during household activities compared to sedentary behavior and physical activity, and another review [61] reported that wearable devices were less accurate outside of laboratory environments.

**Sedentary (inactive) minutes.** One review [60] evaluated the accuracy with which Fitbit devices detected inactive minutes. This review synthesized data for nine distinct Fitbit models utilizing wrist, ankle, and torso wear locations. When benchmarked against clinical-grade accelerometers (e.g., ActiGraph), the authors concluded that Fitbits demonstrated high accuracy in detection of sedentary minutes, with error rates typically below 10%. Errors were largely found to be in the direction of underestimation; only one study included in their review observed overestimation of sedentary time.

### Energy expenditure (EE)

Fourteen reviews evaluated the accuracy of consumer wearable-assessed estimates of EE, inclusive of 37 devices (see S8 Appendix for detailed reporting at the device level). Most reviews concluded that the accuracy of EE estimation—regardless of device—was either low or variable (e.g., high for some populations or use settings but low in others).

**Determinants of accuracy.** Specific details about populations with diminished accuracy for EE were reported by two reviews [41,43]. One [41] reported that Apple Watch overestimated total EE in female participants, but underestimated it in male participants, and found that EE MAPE was between 14.07% and 210.84%, with larger errors at higher BMI and for males. Examining studies using Apple Watch models, one review [43] found that samples with more women yielded lower MAPE for EE than samples with more men. They also observed significant differences in MAPE due to variation in age and BMI.

Six reviews reported differences in accuracy based on activity type and data acquisition setting. One review [41] reported that Apple Watch tended to overestimate EE, with the greatest error during walking and lowest error during cycling. Another [47] reported that compared to the Oxycon Mobile device (a laboratory-grade tool), the Fitbit Classic underestimated EE for cycling, raking, laundry, and treadmill walking, but overestimated EE for carrying groceries; they also identified inaccuracies in EE estimation for resistance exercises by Fitbit Flex, Jawbone Up24, Nike+ Fuelband SE, Misfit Shine, and Polar Loop. Another [59] found that when benchmarked against indirect calorimetry, devices overestimated EE during walking (Fitbit Ultra), in laboratory-based activities (Fitbit Zip), during inactivity, aerobic, resistance exercise (Fitbit Flex), and running (Jawbone Up). Alternately, another review [40] reported that Fitbit devices had higher validity in measuring EE during ambulatory activities (defined as running and walking) compared to lifestyle activities (defined as activities of daily living such as chores, which were sometimes combined with ambulatory activities). Another [42] reported adequate estimation of EE during treadmill running and overestimation during light and moderate activities for the Fitbit Charge HR. Differences based on where in the body wearable devices are placed were also observed [60]: those placed on the torso had measurement error lower than 3% more than 60% of the time, whereas wrist placement resulted in error of greater than 3% more than 60% of the time.

**Reliability.** Though limited, available data suggested that device measurements were somewhat inconsistently correlated with one another: in one review [34], across 5 studies correlation coefficients were reported for 16 of 50 inter-device comparisons of EE estimates. Among these, correlations were weak for 13% of comparisons, moderate for 6% of comparisons, strong for 6%, and very strong for 75%.

### Sleep metrics

**Total sleep time (TST).** Five reviews included data relevant to TST accuracy, spanning 14 wrist-worn devices (see S9 Appendix for detailed reporting at the device level). Comparators used for benchmarking included polysomnography (PSG), actigraphy, and sleep diaries or logs. The most detailed reporting on accuracy was available for Fitbit and Jawbone devices. Compared to PSG, Fitbit devices were consistently found to overestimate TST [59,60,62,63], with the lowest rates of overestimation identified for Fitbit Alta. Jawbone device estimates were also found to deviate from PSG-assessed TST [59,60,62,63]. When actigraphy-assessed TST was used as a benchmark, Fitbit devices were found to provide overestimates while Jawbone device assessments were acceptably consistent with actigraphy [62,63]. Only one review [63] addressed determinants of accuracy, and reported that differences between Fitbit Ultra and PSG were greater in groups with sleep-disordered breathing, as well as differences in measurement accuracy between age groups.

**Sleep efficiency (SE).** Five reviews evaluated SE, covering 12 distinct devices (see S10 Appendix for detailed reporting at the device level). Fitbit and Jawbone devices were the most frequently reported on. Fitbit devices were found to overestimate SE compared to PSG in “normal mode”, and to underestimate SE in “sensitive mode”; similar results were obtained when compared against actigraphy, with slight overestimation in “normal mode” and a larger underestimation in “sensitive mode” [60,62,63]. Jawbone and Jawbone Up were also found to overestimate SE compared to PSG [59] although another review [63] reported minimal differences in data measurements between actigraphy and Jawbone Up. Fitbit and Garmin devices demonstrated varying accuracy as compared to sleep logs [59,62]. Fitbit Versa and Fitbit Charge 2 slightly underestimated SE compared to the sleep scope [62]. Only one review [63] evaluated determinants of accuracy, and found differences in accuracy across age groups.

**Sleep onset latency (SOL).** Five reviews evaluated SOL, spanning 12 distinct devices (see S11 Appendix for detailed reporting at the device level). Benchmarking comparators reported on included PSG, actigraphy, an EEG-based sleep scope, and a sleep log. The largest volume of information was available for Fitbit and Jawbone brand devices and, overall, there was heterogeneity in findings reported. Fitbit and Jawbone devices’ accuracy was found to vary depending on the comparator used (actigraphy vs. home PSG), mode (normal vs. sensitive), as well as the population being evaluated [38,60,62]. For example, benchmarked against actigraphy, Fitbit devices overestimated SOL in normal and sensitive mode, with sensitive mode having slightly lower correlations with actigraphy [62,64]. The Fitbit Alta HR was found to overestimate SOL compared to sleep logs, while several Fitbit devices underestimated SOL compared to the sleep scope. Jawbone Up was found to overestimate SOL compared with actigraphy [63]. Other devices reviewed were evaluated as having lower accuracy: PSG-assessed SOL was uncorrelated with estimates provided by the Withings Pulse O2, and intraclass correlation (ICC) between PSG and the Zero Wireless system were low-to-moderate (ICC range: 0.42-0.67) [38].

**Wake after sleep onset (WASO).** Four reviews assessed the accuracy of WASO estimates. These spanned 11 devices, all of which were Fitbit or Jawbone brands (see S12 Appendix for detailed reporting at the device level). Compared with PSG and actigraphy, Fitbit devices underestimated WASO to various extents in normal mode and overestimated WASO in sensitive mode [62]. Jawbone device-assessed WASO also deviated significantly from PSG and actigraphy [59,63]. Compared to actigraphy, Fitbit-assessed WASO and a self-reported sleep log were not significantly correlated [62]. One review [63] evaluated determinants of accuracy and found that individuals with sleep-disordered breathing and differences in ages had differing levels of accuracy between Fitbit Ultra and PSG.

### Other sensor data

Seven reviews evaluated accuracy of other types of sensor data with potential relevance to chronic illness management: distance traveled, physical activity duration, time spent in moderate-to-vigorous-intensity physical activity (MVPA), seizure activity, ECG signal quality, and respiratory rate (see S13 Appendix for reporting at the device level). These sensors were largely discussed in reviews in the context of low or variable accuracy. For example, four reviews [54,64–66] addressed physical activity duration, all of which noted either variable or low accuracy for this metric when compared with other types of monitors. A notable exception was one review evaluating ECG signal quality, which reported that Apple Watch Series 4 was found to have high correspondence in ECG signal quality compared with a 12-lead ECG [66].

### Overall conclusions about categories of devices

**Fig 5:**
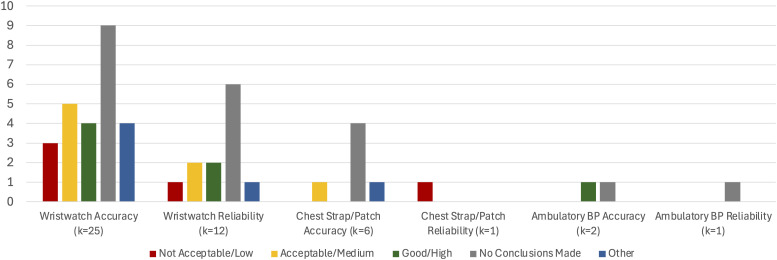
Overall conclusions about accuracy, validity, and reliability of consumer wearable devices. “Accuracy” was determined based on evaluative statements made by reviews about the accuracy or validity of data collected through devices compared to a benchmarking metric. “Reliability” was evaluative statements made about any form of reliability (e.g., test-retest reliability).“Other” is used when a review drew evaluative conclusions not readily categorizable into low/medium/high categories, such as concluding mixed results across device brands or types of sensors.

**Fig 6:**
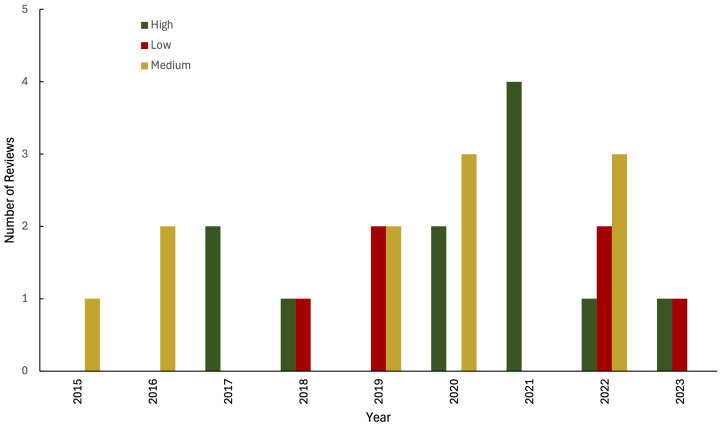
Reviews’ conclusions about wearable accuracy over time. Chart is based on k = 28 reviews drawing low/medium/high conclusions.

Many reviews evaluated the accuracy of overarching categories of wearable devices (e.g., focusing on wristwatches or chest straps in general rather than specific brands or devices). The majority of reviews that evaluated categorical accuracy eschewed making evaluative conclusions, and there was considerable heterogeneity in conclusions when any were drawn (Fig 5). There is no discernible trend in the general conclusions drawn (e.g., low, medium or high accuracy) over time (year of publication) (Fig 6).

### Clinical utility

**Fig 7:**
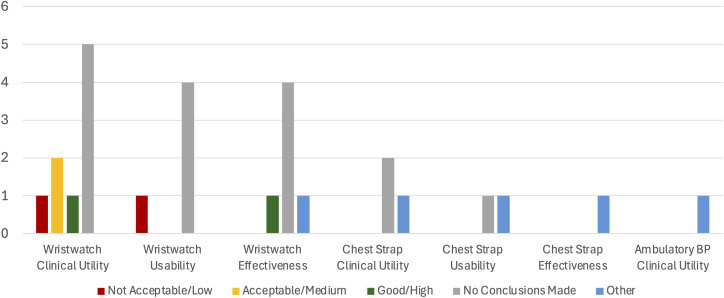
Overall conclusions about clinical utility, usability, and effectiveness of consumer wearable devices. “Utility” was defined as an evaluation of the wearable’s usefulness to clinical assessment, diagnosis, symptom monitoring, or symptom management. “Usability” encompassed evaluative statements about the usability, acceptability, or feasibility of patient use of devices. “Effectiveness” was defined as effectiveness at improving clinical outcomes of interest to the review, where applicable (e.g., for a review focused on a chronic pain population, effectiveness of the device at addressing chronic pain-related concerns). “Other” is used when a review drew evaluative conclusions not readily categorizable into low/medium/high categories, such as concluding mixed results across device brands or types of sensors.

Several reviews evaluated clinical utility of devices categorically (e.g., wristwatches as a whole, chest straps as a whole). Most eschewed making overall conclusions, summarized in Fig 7.

#### Accessibility.

Barriers to accessibility identified across k = 4 reviews included cost [51,57], use complexity or low technology literacy [51,57], and availability [55]. One review reported identifying no barriers to accessibility [67].

#### Adherence.

Of k = 4 reviews evaluating adherence, two found consumer wearable devices to yield acceptable adherence [41,68]. The remaining two found that factors such as data loss, data gaps of long duration, qualitative feedback indicating mixed adherence, and lack of support materials about the devices available to participants, undermined confidence in adherence among the studies they reviewed [56,69].

#### Diagnostic utility.

Six reviews reported on the utility of consumer wearable devices for clinical diagnosis, all of which focused on cardiac conditions. For the most part, these reviews drew favorable conclusions about the devices evaluated. Two reviews [52,53] concluded that Apple Watch was adequate for diagnosing likely atrial fibrillation. Another review [52] found that likely atrioventricular block was adequately diagnosable by Apple Watch and Fitbit devices, that likely bradycardia was adequately diagnosable by Apple Watch, and that the Fitbit Charge HR was adequate for diagnosing likely atrial flutter. Additionally evaluated devices included KardiaBand (evaluated as adequate for diagnosing likely atrial fibrillation), Samsung devices of unspecified series (evaluated as adequate for diagnosing likely atrial fibrillation), and iRhythmZio (evaluated as adequate for diagnosing likely atrial fibrillation and sustained atrial tachycardia) [53]. One additional review [35] noted adequate diagnostic utility of wearable devices for atrial fibrillation and arrhythmia detection, but did not specify the devices from which this conclusion was drawn.

#### Effectiveness for addressing clinical outcomes of interest.

The limited evidence addressing effectiveness of wearable use on clinical outcomes of interest all evaluated clinical effectiveness of wearables for modifying physical activity. Specifically, seven reviews [37,54,56,58,68,70,71] operationalized physical activity outcomes as increased step count (no other outcomes were represented). All seven reviews found evidence of effectiveness for wearable devices as an intervention that can increase physical activity. Two reviews drew conclusions specifically about a clinical population: one concluded that wearable tracking of physical activity combined with exercise training increased physical activity in hospitalized patients with ischemic stroke [54], and one concluded that wearable tracking of physical activity significantly improved self-reported pain-free walking ability [71].

### Identified areas with no or very little data

#### Accuracy.

No device-level information about accuracy was found for the following biometrics: oxygen saturation (O2Sat), electrodermal activity (EDA; also referred to as galvanic skin response or GSR), or flights of stairs climbed. No reviews addressed accuracy of skin temperature assessments, although one review [64] reported data missingness, which may impact overall accuracy. That review found that the Basis Peak wristwatch had an 11.12% missed data rate over three nights of measuring skin temperature compared to the SOMNOscreen polysomnography (PSG) device.

One systematic review and meta-analysis reported information about accuracy of detecting arrhythmias other than atrial fibrillation by smartwatches [63]. That review identified only two studies containing information about arrhythmias other than atrial fibrillation. Both studies were prospective, and the review authors concluded that the available evidence was too limited to draw conclusions.

Two reviews evaluated the accuracy of V02 max detection, both of which produced incomplete information. One [48] reported that the Fitbit Charge 2 had a MAPE of 9.14% for V02 max, a value that would typically be considered low (corresponding with high accuracy), but did not specify the comparators used for benchmarking. Another [61] similarly reported a MAPE of <10% for consumer health wearables as benchmarked to respiratory gas analysis, but did not specify which consumer health wearables were evaluated in this analysis.

No information about determinants of accuracy (populations or settings with evidence of diminished accuracy) was identified for the following biometrics: HRV, atrial fibrillation, 02Sat, V02max, skin temperature, sedentary or inactive minutes, SOL. No information about reliability was reported for the following biometrics: HRV, atrial fibrillation, 02Sat, V02max, skin temperature, sedentary or inactive minutes, TST, SE, SOL. For all sleep metrics, the only determinant of accuracy evaluated was age [63].

#### Acceptability.

Only one review [55] reported explicitly on patient, user, or participant acceptability, but the authors did not draw conclusions about device acceptability or usability. No reviews were found to evaluate acceptability of patient use of consumer health wearables from the perspective of clinicians.

#### Adverse events and adverse effects.

None of the 42 reviews were found to report on adverse events, adverse effects, negative outcomes, or undesired effects of wearable devices.

## Discussion

This systematic umbrella review synthesized findings from 42 published reviews, evaluating the accuracy, validity, reliability, and clinical utility of consumer health wearables, with a focus on their application in outpatient self-monitoring for post-infection syndromes such as PASC. Our findings illuminate areas of strength in wearable-assessed biomarker detection relevant for self-management of chronic conditions, while also revealing substantive gaps in the literature and considerable room for improvement in the accuracy, reporting consistency, and evaluation of population-based differences in performance of wearable devices.

### Strengths of consumer health wearables for clinically relevant self-monitoring

Several areas of strength were identified. Of all biometrics included in this review, the greatest volume of evidence was observed for HR monitoring. Wrist-worn devices were the most frequently reviewed, and Apple Watches received the most consistently high accuracy ratings for HR detection, a metric that may be useful to the many patients with post-acute infection syndromes who have dysautonomia [14,72]. Other wrist-worn devices available at lower price points (e.g., Fitbit and Garmin brand devices) received generally favorable but more heterogenous accuracy evaluations that varied based on the setting and context of use [42,47]. Sensors using red spectrum light – found in most wrist-worn devices including Apple Watch – also yielded reductions in accuracy for individuals with darker skin. These findings suggest that many commercial wearable devices are likely acceptable for HR monitoring, but that patients interested in tracking activity-driven patterns in their heart rate, as well as those with dark skin tones, may be best advised to use chest-strap devices (rather than wristwatches). Although chest-strap devices were included in only a small number of reviews, the available review-level evidence suggests that they have the highest correspondence with ECG.

Apple Watches also received the most consistently high accuracy ratings for AF detection across multiple reviews. Of note, several models of Apple Watch have received U.S. FDA clearance or qualification (distinct from full FDA approval) for AF detection. Further, there appears to be consensus across reviews that wrist-worn wearables have adequate accuracy for detecting AF. This functionality may be useful for many PASC patients: a recent meta-analysis identified an increased risk of AF events in individuals who have recovered from COVID-19 compared to the general population [73], and incidence of arrhythmias among those with cardiovascular complications from COVID-19 has been estimated at 18%, with AF being the most common arrhythmia [74].

### Measuring up: Weak evidence and evidence of weakness in consumer wearables

Any appraisal about clinically relevant uses for consumer health wearables should rely on high-quality evidence. It is therefore notable that the quality of evidence identified by this umbrella review was highly variable. Out of 16 possible criteria used by the AMSTAR 2.0, the average number that met criteria for “yes” or “partial yes” was only 6, and only 38% of reviews included any risk of bias assessment. Relatedly, for most of the accuracy and validity indices extracted by this umbrella review, reporting was highly inconsistent. This made any meaningful quantitative aggregation of evidence across reviews virtually impossible. The absence of a common standard or lexicon for high-quality health wearables research, at the level of individual studies as well as reviews, poses a major obstacle to gathering high-level evidence on wearable-based measurement in health contexts. The data that were aggregated descriptively indicate that accuracy of wearable-assessed metrics varies greatly depending on the device, the biometric, and the context of wear.

Wearable devices are sometimes marketed as an efficient and effective way to track energy expenditure, whether for behavioral weight management or physical activity modulation. However, EE estimates were rarely accurate, showed MAPEs exceeding 200% in some populations [41], and were reported to be less accurate for males and individuals with higher BMI. This suggests poor basis for using wearables to track calories or other types of EE. Additionally, little evidence was available for blood oxygen saturation or V02 max, two metrics likely to be of high interest to patients with post-infection syndromes featuring respiratory symptoms. The two reviews evaluating V02 max reported incomplete information for this biometric, but suggested high error rates in measurement from the data that were available.

Other gaps in the literature included limited data about acceptability from the perspective of providers, which may represent a significant barrier to implementation. Health information technology-related stress is a well-established predictor of physician burnout [75,76]. Although this body of research has primarily focused on electronic health record (EHR) systems rather than adjunctive technologies such as wearables, it foreshadows broader implementation challenges that warrant targeted research. Potential barriers to provider acceptability include increased workload associated with reviewing additional streams of patient health data, challenges integrating wearable-assessed data into EHR systems, and concerns related to the privacy and security of these health data. Novel applications of blockchain technology may be useful both to the eventual integration of wearable-assessed data into EHR systems, and to ensuring the secure protection of these data [77].

#### Absence of data on adverse effects.

This review was unable to identify any review-level reporting of adverse events or adverse effects at all, including an absence of evidence for these effects. Several constraints hamper the assessment of adverse effects in consumer wearables research. Adverse effects assessment in studies of wearable technologies has never been systematized, and no articles in this review describe any formal method for AE assessment. This suggests that, as in other research areas [78,79], studies typically rely on passive monitoring (i.e., adverse events are only recorded if a participant volunteers one), rather than specific solicitation. And yet, passive monitoring under-counts AEs by up to a factor of 20 [80] compared to AE solicitation. For U.S. FDA-regulated research, which includes the investigation of medical devices, current AE assessment frameworks rely on medical and pharmacological lexica such as the Medical Dictionary for Regulatory Activities (MEDdra; [81]) or Common Terminology Criteria for Adverse Events (CTCAE; [82]). However, reliance on such criteria are known to systematically under-count AEs in behavioral and lifestyle interventions [ 83,84], suggesting that these lexica may not be sufficient for research on consumer wearables.

Primary studies suggest that misinterpretation of wearable-assessed data may result in increased health anxiety for individuals with a chronic condition, among other possible untoward psychological and behavioral effects [84,85]. For patients with PASC and post-acute viral syndromes specifically, effective symptom monitoring is a high priority. A recent systematic review of interoception in individuals with a chronic condition found that high interoceptive sensibility (an individual’s belief in their ability to detect and regulate interoceptive signals) is associated with lower symptom severity, but that interoceptive accuracy is generally lower for this population [86]. Wearables may therefore be regarded by patients as crucial tools for supporting improved interoceptive sensibility, especially when symptomatic or following exertional activity. Thus, individuals who have a chronic condition are especially vulnerable to harms such as health anxiety and symptom perseveration that can result from inaccurate data or inaccurate contextualization of data. Compounding this problem, many of the core symptoms (e.g., fatigue) that patients with post-acute infection syndromes track are multifaceted and not directly reducible to wearable-assessed biometrics. To facilitate comprehensive AE assessment, formative research is needed to identify AEs that may be eventuated by consumer health wearables. Doing so would help to identify risk factors for specific AEs, as well as modifications that can attenuate risk of harms while maximizing the benefits of using these devices.

#### Assessment of clinical utility is an unmet need.

Very few reviews evaluated how well wearable-derived data support diagnosis, management, or improvement of patient outcomes. Further, while review-level evidence was identified for the utility of wearables in assessing and monitoring atrial fibrillation, no reviews evaluated other types of cardiovascular symptoms more common in post-infection syndromes (e.g., postural orthostatic tachycardia syndrome, other types of dysautonomia, other arrhythmias). Evaluations of effectiveness for addressing clinical outcomes were restricted to increasing physical activity. Only two reviews focused on increasing physical activity in clinical populations, indicating limited existing evidence that aligns with non-progressive rehabilitation, the leading behavioral treatment recommendation for PASC and many other post-acute infection syndromes [74,87]. Although consumer health wearables appeared to be effective at driving increases in physical activity, this review is limited in its capture of this literature because reviews that primarily focused on interventions beyond wearables were excluded; accordingly, reviews that addressed wearables and physical activity interventions together are not represented here. Recent evidence contradicting our findings exists: for example, one recent study [88] found that physical activity actually declined for those using consumer health wearables. Data about clinical utility [25]—when and under what circumstances consumer wearables are a useful component of a self-management plan—were otherwise observed in this review to be lacking. These are needed for the development of evidence-based recommendations for their integration into outpatient care.

#### Equity and underrepresentation: Wearables do not work equally well for everyone.

This review identified a concerning lack of review-level evaluation of device performance across diverse demographic groups. While age (reported in 74.5% of reviews) and gender (reported in 69% of reviews) were frequently addressed, race and ethnicity were rarely examined, appearing in only 7% (k = 3) reviews and fewer than 5% (k = 2) reviews, respectively. When demographic factors were examined, findings often suggested diminished performance for some biometrics and devices based on these characteristics—for example, the finding that photoplethysmography (PPG) sensors (used commonly for HR measurement in wristwatch devices) may be less accurate in individuals with darker skin tones [44]. Socioeconomic status was also infrequently included (14% or k = 6 reviews), despite its clear relevance to wearable device access. Many wearable devices depend on smartphone pairing and reliable internet connectivity, which may systematically exclude individuals with lower incomes or those living in rural areas. These equity issues are highly intersectional, given that individuals from lower income backgrounds are disproportionately represented among historically underrepresented racial and ethnic groups. Together, these gaps raise concerns about the generalizability and equity of the current evidence, and highlight a need for more inclusive primary studies and consistent reporting of demographic characteristics in future research.

Further, most reviews synthesized studies conducted in healthy populations, with only 7 of 42 reviews specifically targeting populations with a defined clinical or morbidity characteristic. For most biometrics, there was an absence of evidence for clinical vs. healthy populations, and where evidence was available, it indicated that performance in healthy individuals did not generalize to individuals with certain morbidity characteristics. Specifically, two morbidities that commonly affect patients with post-acute infection syndromes – slow gait and respiratory problems – were found to correspond with deviations in accuracy for biometrics across multiple devices [47,57,61]. Older and younger age were also associated with deviations in accuracy for several indices across multiple devices. This indicates a need for primary studies investigating performance in the presence of possible health confounds, including medical conditions and the medications used to manage them.

#### Summary of gaps in evidence.

Despite the large number of primary studies of consumer health wearables, and a correspondingly high number of reviews written about this primary literature, those reviews were found to have inconsistent quality, to be highly skewed towards healthy populations, and lacking in demographic representation. Despite a high volume of research, the aggregations of this research have yielded only limited evidence upon which to make confident recommendations about clinically relevant self-monitoring, especially for chronic health conditions such as PASC. Gaps in the evidence identified by this umbrella review, and corresponding recommendations, are summarized in Table 3.

**Table 3:** Gaps in the evidence and corresponding recommendations.

Identified Gaps in Evidence	Recommendation
**Demographic underrepresentation** (e.g., limited reporting of race/ethnicity, minimal focus on older adults), with evidence of variability in accuracy based on demographic characteristics where information is available	-Future validation studies should prioritize diverse recruitment across race, ethnicity, socioeconomic status, age, and geography to ensure generalizability and identify demographic-based differences in measurement accuracy -Future reviews should stratify evidence according to demographic variables, to facilitate the development of review-level evidence with adequate demographic representation
**Heterogenous evidence for measurement accuracy**, with inconsistent reporting and wide variability by biometric, device brand, and device series	-Future studies should describe device brand, device generation/series, and biometric(s) assessed as reporting conventions -Future studies of accuracy should adopt standardized benchmarking protocols (e.g., consistent comparators, reporting of error metrics, inclusion of reliability testing) to improve comparability across studies for future reviews
**Insufficient evidence for clinical populations.** Most validation studies are restricted to healthy populations, leaving individuals with any medical diagnosis largely unrepresented	-Primary studies investigating device accuracy or performance among individuals with clinical diagnoses and symptoms are needed -Future reviews of such studies should evaluate any medical factors that may be accuracy confounds or otherwise interfere with device performance
**Narrow scope of outcomes studied**, resulting in weak evidence for the utility of wearables for health management	-Expand clinical effectiveness research to outcomes directly relevant to post-infection syndromes, including fatigue, sleep, autonomic dysfunction and cognitive impairment -Expand effectiveness research to include wearable-assisted treatments (e.g., for implementation of activity or exertion medical recommendations) -Encourage long-term, real-world studies to evaluate sustained accuracy, reliability, safety, and potential unintended effects
**Absence of data on adverse and undesired effects**	-Actively solicit AEs from participants to mitigate underreporting associated with reliance on volunteered information -Conduct formative research to identify AEs that may be eventuated by consumer health wearables, particularly for individuals who have a chronic health condition

### Limitations and the role of living reviews

This umbrella review was pre-registered and employed comprehensive search and extraction procedures. Nonetheless, several limitations must be acknowledged. Variation in how devices were named or categorized (e.g., unspecified Fitbit models), as well as in the level at which data were reported (e.g., sometimes at the device level and sometimes at the level of type of wear, such as consumer wristwatches overall) limited our ability to draw fine-grained comparisons across studies. Relatedly, this review excluded non-consumer, medical-grade devices, which may have yielded different performance profiles. This review also did not cover algorithms applied to biometric assessments (e.g., differences among algorithms used to estimate HRV) or data transmission, which are other key aspects of these technologies that bear upon accuracy and usability. Although we extracted reported data about acceptability broadly, we did not specifically target data privacy or security in this review. Given the role of these concerns in wearable use, this is another important limitation of this review.

As noted previously, this umbrella review employed broad search and inclusion criteria given that PASC only emerged as a recognized diagnosis in 2020 and limited data for individuals with others post-acute viral syndromes exist. Reflecting this paucity of data and the broader tendency for wearable research to focus on healthy populations, only 7 of 42 reviews examined populations with a clinical or morbidity characteristics at all. Accordingly, this review provides extrapolated findings with relevance to PASC and post-acute infection syndromes and should not be interpreted as direct evidence.

This synthesis was also constrained to review-level literature, potentially missing recent empirical studies not yet captured in systematic reviews, or captured in systematic reviews after the article search used in the present analysis. Smart rings such as the Oura ring increased in popularity while this review was being conducted, and are minimally represented in this umbrella review due to an absence of review-level data about smart rings that went beyond theoretical discussion at the time of our article search. A recently published meta-analysis [89] of smart rings found acceptable accuracy for smart ring detection of HR, inconsistent performance for sleep-related metrics, and insufficient evidence to support evaluation of other cardiovascular biometrics (e.g., HRV, oxygen saturation), situating the current evidence for smart rings as comparable to the evidence for smart watches identified by this umbrella review.

Given the rapid pace of development in wearable technologies, this review has placed extracted data on the Open Science Network to enable consistent updating as a living review. Current results illuminate overall patterns in findings (and non-findings) in the literature that remain important targets for the next wave of research, and that can illuminate areas of caution about the validity of data for consumers. Specifically, a corpus of literature has emerged evaluating a variety of interventions, populations, and outcomes by means of wearable devices and device-classes that are included in this umbrella review. As such, the data herein provides a point of reference about the status of such evidence (e.g., error rates of EE may suggest caution in interpretation of studies that exclusively relied on wearable-assessed EE). Updates to the analyses presented here as a living review will be crucial to this field as new technologies are introduced and evidence about their performance continues to accumulate.

Queriable databases that maintain and aggregate information about wearable device performance, such as the Stress in Action Wearables Database [90], are also valuable resources for researchers and consumers alike, given the speed at which they can be updated. Databases such as this, and living systematic umbrella reviews such as ours and others [91,92] serve complementary purposes: databases alone do not provide a systematic means of evaluation and interpretation of available information, and umbrella reviews lag behind the timeliness of a consistently updated database (owing to the complexity of procedures required to extract and bring into relationship diverse and differently organized findings).

### Are we there yet? Conclusions and recommendations about consumer health wearables for outpatient health monitoring

Results from this review suggest that there is not sufficient evidence to recommend the use of consumer wearables for self-monitoring to the millions of individuals living with PASC and other post-acute infection syndromes. Evidence suggests that wearables are suitable for atrial fibrillation detection and for HR monitoring when patients are comfortable with margins of error. For both biometrics, the largest volume of evidence supporting acceptable device accuracy exists for Apple Watch, possibly supporting a current recommendation for this brand in particular—although this may be partially a function of publication bias in what devices are most commonly included in review-level analyses. Little evidence was available for biometrics that may be of interest to individuals with shortness of breath, such as blood oxygen saturation or V02 max. For biometrics relevant to activity pacing, there was either limited evidence or clear evidence of weakness: EE was found to have low accuracy overall, and step counts were found to have low accuracy among individuals with slow gait speeds, an abnormality that frequently occurs in PASC [93]. There was no review-level evidence to support the idea that detection or self-monitoring via wearables leads to improved downstream outcomes (e.g., reduced morbidity, better self-management, reduced healthcare utilization).

**Fig 8:**
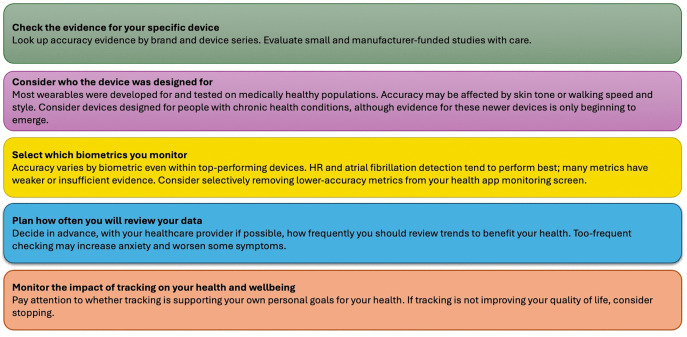
Five recommendations for informed use of consumer wearables for outpatient health monitoring.

How do we reconcile this with a primary study finding that overwhelmingly, individuals with PASC who are already using a wearable perceive it to be helpful [23]? Or the fact that treatment guidelines are well-aligned with the biometrics that consumer wearables putatively track – for example, the recommendation to undertake exercise guided by subjective toleration and biometrics such as heart rate during exercise, blood oxygen saturation, and heart rate recovery in the first minute after exercise termination [74,87,94]? Informed use – that is, use guided by an understanding of what brands and biometrics have stronger versus weaker evidence for accuracy, and activities or contexts of use that can diminish the accuracy and validity of data – offers the best path forward for patients who are already using or considering a wearable device. Fig 8 provides practical recommendations for informed use guided by findings from this review. In addition, new-to-market devices that are designed for use by people who have a chronic illness (contrasted with most commercial devices which were designed with a healthy user in mind), may be an important future direction. However, as of the submission of this article, peer-reviewed research for such technologies (e.g., Visible) [95] is not yet available.

There is a clear need both for more primary data and more review-level aggregations of evidence focused on populations other than those who are medically healthy. In the meantime, the public health implication of the results of this systematic umbrella review suggests that caution should be taken in recommending consumer wearable devices to individuals who have a chronic condition.

## Supporting information

S1 Appendix PubMed Search Strategy for Publication. (DOCX)

S2 Appendix Included reviews and devices evaluated (k = 42). *Note. CNI* = Could Not Identify and is used when the review did not report on the total number, or if conflicting information about the total number of included studies was identified in the article. *NR* = Net Reported and is used to indicate that the information was not found in the review. ^ is used for device count for reviews for which the generation or series was not always specified (e.g., “Fitbit”), as this number may therefore be an undercount of the actual distinct devices represented by the review. *Included devices* represent consumer health wearable devices reviewed only; medically prescribed devices were excluded from this review and are therefore not represented. Device names are reported here as reported in the original review. In some cases, device names may have changed since the publication of the reviews included in this article. * indicates series was unspecified for the device. **indicates both series and generation were unspecified for the device.
(DOCX)

S3 Appendix Quality Assessment Heat Map. *Note.* For Statistical Methods and RoB Assessment Categories, appropriate statistical methods were used for RCTs and NRSIs separately using the AMSTAR 2.0 checklist and studies that included both RCTs and NSRIs, a “yes” for one and “no” for the other resulted in a “partial yes” determination from us. “% Y or PY” reflects the percentage of all applicable categories (out of 16 for studies that included a meta-analysis and 13 for studies that did not) that received a designation of yes or partial yes.
(DOCX)

S4 Appendix Risk of Bias Assessment. *Note.* “High” indicates authors concluded a high ROB. “Medium” indicates authors concluded a medium ROB. “Low” indicates authors concluded a low ROB. “Other” indicates that review authors described completing an ROB assessment as part of review procedures but did not report conclusions based on this information.
(DOCX)

S5 Appendix Heart rate (HR) accuracy benchmarking. *Note.* *** indicates that information was not reported by the authors. – indicates that some information was reported, but insufficiently to determine a rating.
(DOCX)

S6 Appendix Atrial Fibrillation Detection accuracy benchmarking. *Note.* *** indicates that information was not reported by the authors. – indicates that some information was reported, but insufficiently to determine a rating.
(DOCX)

S7 Appendix Physical Activity: Steps Taken accuracy benchmarking. *Note.* *** indicates that information was not reported by the authors. – indicates that some information was reported, but insufficiently to determine a rating.
(DOCX)

S8 Appendix Energy Expenditure accuracy benchmarking. *Note.* *** indicates that information was not reported by the authors. – indicates that some information was reported, but insufficiently to determine a rating.
(DOCX)

S9 Appendix Total Sleep Time (TST) accuracy benchmarking. *Note.* *** indicates that information was not reported by the authors. – indicates that some information was reported, but insufficiently to determine a rating.
(DOCX)

S10 Appendix Sleep Efficiency (SE) accuracy benchmarking. *Note.* *** indicates that information was not reported by the authors. – indicates that some information was reported, but insufficiently to determine a rating.
(DOCX)

S11 Appendix Sleep Onset Latency (SOL) accuracy benchmarking. *Note.* *** indicates that information was not reported by the authors. – indicates that some information was reported, but insufficiently to determine a rating. + Authors of this article conducted multiple tests of similarities and differences; here, we have extracted and reported just Bland-Altman analyses as a concise indicator of agreement between the two measurement methods. The authors full results can be found in their supplementary materials at: Appendix A: Supplementary Materials.
(DOCX)

S12 Appendix Wake After Sleep Onset (WASO) accuracy benchmarking. *Note.* *** indicates that information was not reported by the authors. – indicates that some information was reported, but insufficiently to determine a rating.
(DOCX)

S13 Appendix Other Sensor Data accuracy benchmarking. *Note.* *** indicates that information was not reported by the authors. – indicates that some information was reported, but insufficiently to determine a rating.
(DOCX)
