# Supplementary material for: Can consumer wearables support outpatient health monitoring for patients with post-acute infection syndromes? A systematic umbrella review of accuracy, validity, and clinical utility data
Source: PLOS Digit Health. 2026 Jun 8;5(6):e0001124. doi: 10.1371/journal.pdig.0001124 (PMC13245765; doi:10.1371/journal.pdig.0001124)
Supplement: S1 Appendix — (DOCX) [file pdig.0001124.s001.docx]

**S1 Appendix. PubMed Search Strategy for Publication**

| #1 | ((consumer*[tw] AND wearable*[tw]) OR "Wearable sensor*"[tw] OR "Health wearable*"[tw] OR Smartwatch*[tw] OR "Smart watch*"[tw] OR "Smart ring*"[tw] OR "smart jewel*" OR "smart cloth*" OR "smart wear*" OR "Fitness tracker*" OR "activity tracker*" OR "Activity monitor*" OR AliveCor*[tw] OR "Apple watch*"[tw] OR Fitbit*[tw] OR Garmin*[tw] OR "Google Pixel"[tw] OR Kardia*[tw] OR Oura[tw] OR (Samsung Galaxy[tw]) OR Suunto[tw] OR Whoop[tw] OR actigraph*[tw] OR actiwatch[tw] ) |
| --- | --- |
| #2 | **(Review*[ti] OR meta-analys*[ti ]OR "Review" [Publication Type] OR "Systematic Review" [Publication Type] OR "Meta-Analysis" [Publication Type])** |
| #3 | **(Review*[ti] OR meta-analys*[ti ] OR "Review" [Publication Type] OR "Systematic Review" [Publication Type] OR "Meta-Analysis" [Publication Type])** |
| #4 | #1 AND #2 |
