# Supplementary material for: Can consumer wearables support outpatient health monitoring for patients with post-acute infection syndromes? A systematic umbrella review of accuracy, validity, and clinical utility data
Source: PLOS Digit Health. 2026 Jun 8;5(6):e0001124. doi: 10.1371/journal.pdig.0001124 (PMC13245765; doi:10.1371/journal.pdig.0001124)
Supplement: S2 Appendix — Note. CNI = Could Not Identify and is used when the review did not report on the total number, or if conflicting information about the total number of included studies was identified in the article. NR = Net Reported and is used to indicate that the information was not found in the review. ^ is used for device count for reviews for which the generation or series was not always specified (e.g., “Fitbit”), as this number may therefore be an undercount of the actual distinct devices represented by the review. Included devices represent consumer health wearable devices reviewed only; medically prescribed devices were excluded from this review and are therefore not represented. Device names are reported here as reported in the original review. In some cases, device names may have changed since the publication of the reviews included in this article. * indicates series was unspecified for the device. **indicates both series and generation were unspecified for the device. (DOCX) [file pdig.0001124.s002.docx]

**S2 Appendix. Included reviews and devices evaluated (k=42)**

| **Study** | **Type of Review** | **Studies Reviewed** | **Wear-type of Devices** | **Devices Included** | **Device Brand and Series** |
| --- | --- | --- | --- | --- | --- |
| Alam 2022 | Systematic review | 6 | wristwatch, clip-on | 6^ | Accusplit AE120XL, Fitbit**, Fitbit Charge Original, Garmin Forerunner, Nike+ FuelBand, Yamax Sw-200 Digiwalker |
| Alharbi 2019 | Systematic review | 20 | wristwatch, clip-on | 8^ | ADAMO Care Watch, Fitbit Charge HR, Fitbit One, Fitbit Zip*, Jawbone UP*, Misfit Shine, Nike+ Fuelband, Polar A300 |
| Avila 2021 | Systematic review | 9 | wristwatch, chest-strap | 3^ | Polar Electro V800, Polar H10, Samsung Gear 3 |
| Bustos 2021 | Systematic review | 38 | wristwatch, clip-on | 6^ | Apple Watch Series 1 Original, Basis Fitness Wristband B1, Basis Peak, Fitbit Charge 2, Polar Vantage*, Samsung Galaxy S4 (worn on body) |
| Evenson 2015 | Systematic review | 22 | wristwatch, clip-on | 8 | Fitbit Classic, Fitbit Flex, Fitbit One, Fitbit Ultra, Fitbit Zip, Jawbone UP, Jawbone UP24, Polar H10 |
| Evenson 2020 | Systematic review | 32 | wristwatch | 12 | Garmin Forerunner 225, Garmin Forerunner 235, Garmin Forerunner 305, Garmin Forerunner 310XT, Garmin Forerunner 910XT, Garmin Forerunner 920XT, Garmin Vivoactive, Garmin Vivofit, Garmin Vivofit 2, Garmin Vivosmart, Garmin Vivosmart HR, Garmin Vivosmart HR + |
| Feehan 2018 | Systematic review | 67 | wristwatch, clip-on | 8^ | Fitbit Charge*, Fitbit Classic, Fitbit Flex, Fitbit Force, Fitbit One, Fitbit Surge, Fitbit Ultra, Fitbit Zip |
| Fuller 2020 | Systematic review | 158 | wristwatch, clip-on | 18 | Apple Watch Original, Apple Watch Series 2, Fitbit Alta, Fitbit Blaze, Fitbit Charge HR, Fitbit Charge Original, Fitbit Charge 2, Fitbit Flex, Fitbit Flex 2, Fitbit One, Fitbit Surge, Fitbit Ultra, Fitbit Zip, Garmin Fenix 3 HR, Garmin Forerunner 225, Garmin Forerunner 235, Garmin Forerunner 405CX, Garmin Forerunner 735XT |
| Germini 2022 | Systematic review | 65 | wristwatch, clip-on | 30^ | Apple Watch*,  Fitbit**, Fitbit Alta, Fitbit Blaze, Fitbit Charge*, Fitbit Charge HR2, Fitbit Flex, Fitbit One, Fitbit Surge, Fitbit Ultra, Fitbit Zip, Garmin Forerunner 920XT, Garmin Forerunner 225,  Garmin Vivoactiv HR, Garmin Vivoactiv Original, Garmin Vivofit Original, Garmin Vivofit 2, Garmin Vivosmart, Jawbone UP, Jawbone UP2, Jawbone UP24, Mio Fuse, Misfit Shine, Nike FuelBand Original, Nike FuelBand SE, Polar**, Polar A300, Polar Loop, Polar V800, Withings Pulse |
| Irwin 2022 | Systematic review | 8 | wristwatch | 1 | Fitbit Charge 2 |
| Koerber 2022 | Systematic review | 10 | wristwatch, clip-on, chest-strap | 15^ | Apple Watch*, Apple Watch Series 3, Fitbit**, Fitbit Blaze, Fitbit Charge*, Garmin Forerunner 235, Garmin Vivosmart 3, Mio Alpha, Mio Alpha 2, Omron HR 500U, Polar*, Polar OH1, Samsung Gear S, Samsung Gear S2, Xiaomi Mi |
| Kolla 2016 | Systematic review | 7 | wristwatch | 3^ | Fitbit**, Fitbit Ultra, Jawbone UP* |
| Maddocks 2018 | Other | CNI | wristwatch, clip-on | 6^ | Fitbit Flex, Fitbit One, Fitbit Zip, Garmin Vivofit, Jawbone UP24, Omron** |
| McCullagh 2016 | Systematic review | 24 | clip-on | 6 | Omron HJ113-EE, Omron HJ-720iTC, Yamax Digiwalker SW701, Yamax DW-200, Yamax PW610, Yamax SW-200 |
| Moshawrab 2023 | Systematic review | 87 | wristwatch, chest-strap | 11^ | Amazfit 1S, Apple Watch*, Apple Watch Series 6, Motorola Moto 360, Polar H10, Samsung**, Samsung Galaxy Active, Samsung Galaxy Active 2, Samsung Simband, Samsung Simband 2, Shimmer ECG |
| Musa 2023 | Systematic review | 1536 | wristwatch | 11^ | Apple Watch*, Fitbit** Garmin**, Jawbone**, Mio**, Misfit**, Polar**, Samsung**, Under Armour**, Withings**, Xiaomi** |
| Patel 2021 | Systematic review | 14 | wristwatch, etextile (wrist-mounted sensor) | 2 | Fitbit Charge HR, Hidalgo EQ0 |
| Reeder 2016 | Systematic review | 17 | wristwatch | 5 | Basis Peak, Motorola Moto 360, Pebble Classic, Samsung Galaxy Gear, Samsung Gear S |
| Scott 2020 | Systematic review | 71 | wristwatch, headstrap | 6 | Fitbit Charge 2, Fitbit Flex, Jawbone UP, Jawbone UP3, Withings Pulse O2, Zeo Sleep Manager |
| Straiton 2018 | Systematic review | 7 | wristwatch, clip-on, chest-strap | 10^ | Fitbit**, Fitbit Charge*, Fitbit Charge HR, Fitbit One, Fitbit Zip, Jawbone UP*, Misfit Shine, Omron HJ-112, Polar**, Polar A300 |
| Strath 2018 | Systematic review | CNI | wristwatch, clip-on | 4 | Fitbit One, Fitbit Zip, Jawbone UP24, Omron HJ-720ITC |
| Wright 2017 | Systematic review | 7 | wristwatch, clip-on | 8^ | Fitbit Charge HR, Fitbit Flex, Fitbit One, Fitbit Zip, Jawbone UP*, Misfit Shine, Omron HJ-112, Polar A300 |
| Belani 2021 | Meta-analysis | 9 | wristwatch | 2^ | Apple Watch*, Samsung** |
| Chevance 2022 | Meta-analysis | 52 | wristwatch | 7^ | Fitbit Blaze, Fitbit Charge 2, Fitbit Charge 3, Fitbit Charge HR, Fitbit Ionic, Fitbit Surge, Fitbit Versa* |
| Cooper 2018 | Meta-analysis | 9 | wristwatch, clip-on | 6^ | Fitbit**, Kens Lifecorder EX, Kenz Lifecorder Plus, Omron HJ-113, Yamax Digi-Walker SW-200, Yamax Digiwalker 700B |
| Elbey 2021 | Meta-analysis | 9 | wristwatch | 5^ | Apple Watch*, Apple Watch Series 3, Fitbit SW, Samsung Gear Fit2, Samsung Simband |
| Haghayegh 2019 | Meta-analysis | 22 | wristwatch | 10 | Fitbit Alta, Fitbit Alta HR, Fitbit Charge HR, Fitbit Charge 2, Fitbit Classic, Fitbit Flex, Fitbit One, Fitbit Surge, Fitbit Ultra, Fitbit Versa Original |
| Leung 2022 | Meta-analysis | 29 | wristwatch, clip-on | 14 | Fitbit Alta, Fitbit Alta HR, Fitbit Blaze, Fitbit Charge HR, Fitbit Charge HR 2, Fitbit Charge Original, Fitbit Charge 2, Fitbit Classic, Fitbit Flex, Fitbit Flex 2, Fitbit One, Fitbit Surge, Fitbit Ultra, Fitbit Zip |
| Molina-Garcia 2022 | Meta-analysis | 14 | wristwatch | 12 | Fitbit Charge 2, Garmin Fenix 3, Garmin Fenix 5X, Garmin Forerunner 230, Garmin Forerunner 920XT, Polar A300, Polar FT40, Polar F6, Polar F11, Polar RS300X, Polar S410, Polar V800 |
| Nazarian 2021 | Meta-analysis | CNI | wristwatch | 6^ | Apple Watch*, Huawei Honor Band4, Huawei Honor Watch, Huawei Watch GT Samsung GearFit2, Samsung Gear S3 |
| VanRemoortel 2012 | Meta-analysis | 134 | wristwatch, clip-on | 2 | Nike+ (iPod), Polar Activity Watch 200 |
| Zhang 2020 | Meta-analysis | 44 | wristwatch | 12^ | Apple Watch*, Basis Peak**, Fitbit**, Garmin**, Microsoft**, Mio**, Omron**, Philips**, Polar**, Samsung**, Tempo**, TomTom** |
| Ferguson 2022 | Umbrella review | 39 | No devices specified | CNI | No devices specified |
| Sanchis-Gomar 2021 | Umbrella review | CNI | wristwatch, chest-strap, armband | 10 | Apple Watch Series 6, Fitbit Blaze, Fitbit Charge*, Fitbit Charge HR, Garmin Forerunner*, Garmin VivoSmart HR, Polar A360, Polar H7, Scosche Rhythm +, TomTom Spark Cardio |
| Bouzid 2022 | Other | CNI | wristwatch | 1 | Apple Watch Series 4 |
| Harraghy 2019 | Other | CNI | wristwatch | 5^ | Apple Watch*, Fitbit**, Garmin**, Misfit** Polar** |
| Khundaqji 2021 | Other | CNI | wristwatch | 8^ | Apple Watch*, Apple Watch Series 4, Fitbit**, Fitbit Charge HR, Garmin Forerunner 630, Huawei Honor Band 4, Huawei Watch GT, Samsung Gear Fit 2 |
| Li 2016 | Other | 22 | wristwatch | 9^ | Fitbit Surge, Garmin Vivoactive, Garmin Vivofit, Jawbone Up*, Microsoft Band*, Nike Fuelband, Polar Electro, Polar M400, Suunto** |
| Lui 2022 | Other | CNI | wristwatch | 6^ | Apple Watch*, Apple Watch Series 1 Original, Apple Watch Series 2, Apple Watch Series 3, Apple Watch Series 4, Apple Watch Series 6 |
| Nelson 2020 | Other | CNI | wristwatch | 11^ | Apple Watch Series 3, Apple Watch Series 4, Apple Watch Series 5, Fitbit Charge 2, Fitbit Charge 4, Fitbit Versa 2, Garmin Vivosmart 4, Huawei Band 4, Samsung Galaxy*, Samsung Galaxy Fit, Xiaomi Mi Band 4 |
| Rao 2019 | Other | CNI | wristwatch, clip-on, chest strap | 5^ | Fitbit Charge*, Fitbit Flex, Fitbit One, Polar*, Yamax Digiwalker |
| Shei 2022 | Other | CNI | wristwatch | 5^ | Apple Watch*, Fitbit Charge*, Fitbit Classic, Garmin*, Polar V800 |

*Note. CNI* = Could Not Identify and is used when the review did not report on the total number, or if conflicting information about the total number of included studies was identified in the article. *NR* = Net Reported and is used to indicate that the information was not found in the review. ^ is used for device count for reviews for which the generation or series was not always specified (e.g., “Fitbit”), as this number may therefore be an undercount of the actual distinct devices represented by the review. *Included devices* represent consumer health wearable devices reviewed only; medically prescribed devices were excluded from this review and are therefore not represented. Device names are reported here as reported in the original review. In some cases, device names may have changed since the publication of the reviews included in this article. * indicates series was unspecified for the device. **indicates both series and generation were unspecified for the device.
