# Supplementary material for: Can consumer wearables support outpatient health monitoring for patients with post-acute infection syndromes? A systematic umbrella review of accuracy, validity, and clinical utility data
Source: PLOS Digit Health. 2026 Jun 8;5(6):e0001124. doi: 10.1371/journal.pdig.0001124 (PMC13245765; doi:10.1371/journal.pdig.0001124)
Supplement: S3 Appendix — Note. For Statistical Methods and RoB Assessment Categories, appropriate statistical methods were used for RCTs and NRSIs separately using the AMSTAR 2.0 checklist and studies that included both RCTs and NSRIs, a “yes” for one and “no” for the other resulted in a “partial yes” determination from us. “% Y or PY” reflects the percentage of all applicable categories (out of 16 for studies that included a meta-analysis and 13 for studies that did not) that received a designation of yes or partial yes. (DOCX) [file pdig.0001124.s003.docx]

**S3 Appendix. Quality Assessment Heat Map**

**
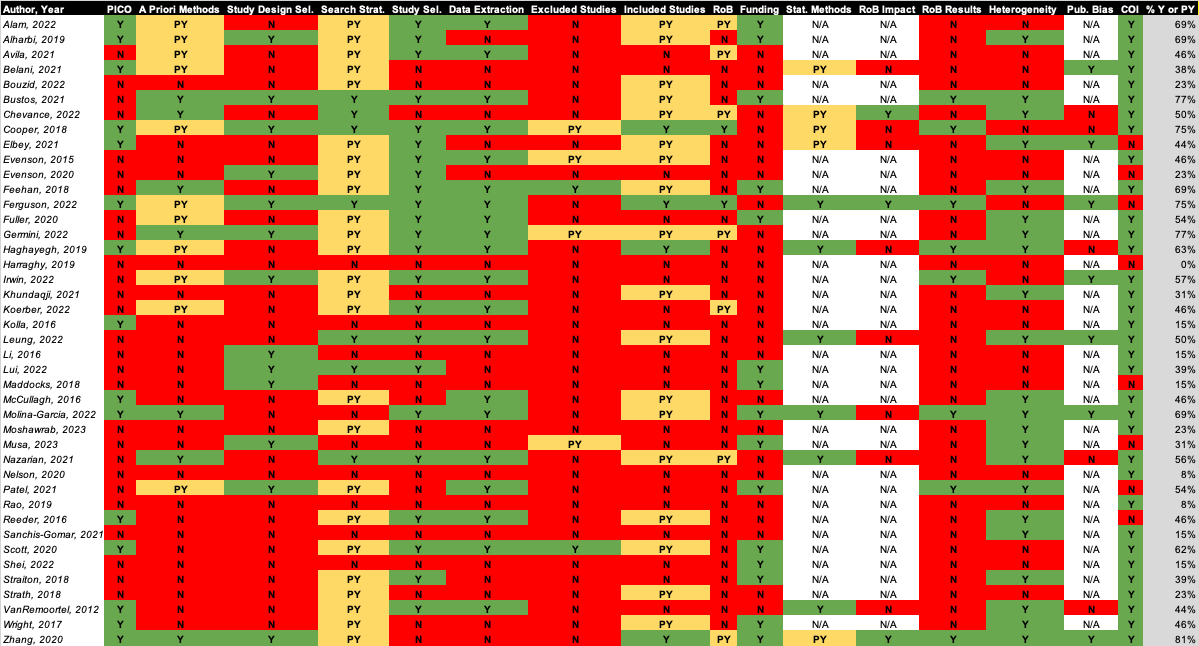
**

*Note.* For Statistical Methods and RoB Assessment Categories, appropriate statistical methods were used for RCTs and NRSIs separately using the AMSTAR 2.0 checklist and studies that included both RCTs and NSRIs, a "yes" for one and "no" for the other resulted in a "partial yes" determination from us. “% Y or PY” reflects the percentage of all applicable categories (out of 16 for studies that included a meta-analysis and 13 for studies that did not) that received a designation of yes or partial yes.
