# Supplementary material for: Can consumer wearables support outpatient health monitoring for patients with post-acute infection syndromes? A systematic umbrella review of accuracy, validity, and clinical utility data
Source: PLOS Digit Health. 2026 Jun 8;5(6):e0001124. doi: 10.1371/journal.pdig.0001124 (PMC13245765; doi:10.1371/journal.pdig.0001124)
Supplement: S4 Appendix — Note. “High” indicates authors concluded a high ROB. “Medium” indicates authors concluded a medium ROB. “Low” indicates authors concluded a low ROB. “Other” indicates that review authors described completing an ROB assessment as part of review procedures but did not report conclusions based on this information. (DOCX) [file pdig.0001124.s004.docx]

**S4 Appendix. Risk of bias (ROB) assessment**

| **Overall ROB** | **Article (Year)** |
| --- | --- |
| High | Molina-Garcia (2022), Fuller (2020), Cooper (2018) |
| Medium | Germini (2022), Patel (2021) |
| Low | Feehan (2018), Nazarian (2021), Alam (2022), Bustos (2021), Irwin (2022), Belani (2021), Ferguson (2022) |
| Other (no conclusions drawn) | Koerber (2022), Chevance (2022), Haghayegh (2019), Avila (2021) |

*Note.* “High” indicates authors concluded a high ROB. “Medium” indicates authors concluded a medium ROB. “Low” indicates authors concluded a low ROB. “Other” indicates that review authors described completing an ROB assessment as part of review procedures but did not report conclusions based on this information.
