# Supplementary material for: Can consumer wearables support outpatient health monitoring for patients with post-acute infection syndromes? A systematic umbrella review of accuracy, validity, and clinical utility data
Source: PLOS Digit Health. 2026 Jun 8;5(6):e0001124. doi: 10.1371/journal.pdig.0001124 (PMC13245765; doi:10.1371/journal.pdig.0001124)
Supplement: S5 Appendix — Note. *** indicates that information was not reported by the authors. – indicates that some information was reported, but insufficiently to determine a rating. (DOCX) [file pdig.0001124.s005.docx]

**S5 Appendix. Heart rate (HR) accuracy benchmarking**

| **Device** | **Benchmarking Device** | **Overall Conclusions (Low, Medium, or High Accuracy)** | **Additional Detail** | **Article (Year)** |
| --- | --- | --- | --- | --- |
| **Apple Watch (Series Unspecified)** | —*** | High interdevice reliability | No additional detail | Fuller 2020 |
|  | Electrocardiography, pulse oximetry, another activity tracker (Not Specified) | High | MAPE ranging from 1% (SD ~1%) to 7% (SD ~11%) | Germini 2022 |
|  | ECG (Not Specified) | High | No additional detail | Koerber 2022 |
|  | Fitbit Charge HR | High | Apple Watch has higher HR accuracy measurements than Fitbit Charge HR | Lui 2022 |
|  | ECG (Not Specified) | High | Apple Watch has significant efficiency in capturing ECG signals, complementing the accuracy of ECGs performed in a doctor’s office, clinic, or hospital room | Moshawrab 2023 |
| **Apple Watch Series 1** | *** | — | HR variability increased as the magnitude of HR measurements increased | Lui 2022 |
| **Apple Watch Series 3** | ECG (Not Specified) | — | HR accuracy decreased in participants with darker skin | Koerber 2022 |
|  | ECG (Not Specified) | High | High accuracy with a mean agreement of 95% | Lui 2022 |
|  | ECG (Not Specified), Fitbit | High | Apple Watch had slightly higher accuracy than Fitbit devices | Nelson 2020 |
| **Apple Watch Series 4** | Polar Vantage V, Garmin Fenix 5, Fitbit Versa | High | Demonstrated highest validity in measuring HR | Lui 2022 |
| **Fitbit Charge (Series Unspecified)** | Electrocardiography, pulse oximetry, another activity tracker (Not Specified) | Low - medium | Mean bias estimated with Bland-Altman method ranged from -6 (SD 10) bpm to -9 (SD 8) bpm | Germini 2022 |
|  | ECG (Not Specified) | — | No HR discrepancies across skin tone | Koerber 2022 |
|  | 12-lead electrocardiograph system | Low | Accuracies were reduced with increasing exercise intensity for both commercial activity monitors; however, the Fitbit charge was far less accurate than the Basis Peak at high-intensity levels, presumably because of factors (e.g., ambient light, sweat, skin contact force) that interfere with the biosensor | Wright 2017 |
| **Fitbit Charge 2** | ECG (ProComp Infiniti T7500M), Polar HR chest strap | Low - medium | Low-medium accuracy | Chevance 2022 |
|  | *** | Low-high reliability | Good reliability on treadmill, poor reliability during hand movement tasks such as dusting | Fuller 2020 |
|  | Electrocardiography, pulse oximetry, another activity tracker (Not specified) | Medium - high | MAPE for HR ranged from 2.4% (SD ~1.5%) to 17% (SD ~20%) | Germini 2022 |
|  | ECG (Not Specified), Polar Chest strap | High | Accurately measured HR 94% | Irwin 2022 |
|  | ECG (Not Specified) | Medium | Slightly underestimated HR across different daily activities | Nelson 2020 |
| **Fitbit Charge 3** | Polar HR chest strap | Low | No additional detail | Chevance 2022 |
| **Fitbit Charge HR** | ECG (Not Specified) | High | No additional detail | Alharbi 2019 |
|  | ECG (Digi- TrakXT & Cosmed C12x & MP150, BioPac System & Q-Stress, Mortara & CASE, GE Healthcare), Polar HR chest strap | Low-High | No additional detail | Chevance 2022 |
|  | Electrocardiography, pulse oximetry, another activity tracker (Not Specified) | Medium - high | MAPE for HR ranged from 2.4% (SD ~1.5%) to 17% (SD ~20%) | Germini 2022 |
| **Fitbit Blaze** | ECG (Quinton 4500 & Not Specified) | Low - medium | No additional detail | Chevance 2022 |
|  | Electrocardiography, pulse oximetry, another activity tracker (Not Specified) | Medium - high | MAPE ranged from 6% (SD 6%) to 16% (SD 18%) for different activities | Germini 2022 |
|  | ECG (Not Specified) | — | No HR discrepancies across skin tone | Koerber 2022 |
| **Fitbit Surge** | ECG (Quinton Q-Stress, version 4.5 & Not Specified) | Medium | No additional detail | Chevance 2022 |
| **Fitbit Versa** | ECG (12-lead CareCenter MD ECG), Indirect calorimetry (TrueMax 2400 Metabolic Measurement System, Parvo- Medics & Jaeger Oxycon Pro) | Low-high | No additional detail | Chevance 2022 |
| **Fitbit Ionic** | ECG (Quinton Q-tel RMS telemetry system) | Medium - high | No additional detail | Chevance 2022 |
| **Garmin Forerunner (Series Unspecified)** | ECG, Apple Watch (series unspecified) | Low | Garmin demonstrated a 0.52 correlation coefficient with ECG data, while Apple watch exhibited a 0.80 | Koerber 2022 |
| **Garmin Forerunner 225** | Polar Chest transmitter | High | All MAPE exceeded 5%  across rest and various laboratory activities | Evenson 2020 |
| **Garmin Forerunner 235** | 12-lead ECG | High | MAPE was 6% at rest, and was higher with increasing intensity, particularly when arm movement was involved | Evenson 2020 |
| **Garmin Vivosmart** | ECG (Not Specified) | High | CC varying widely  across activities and the MAPE exceeding 5% in all cases | Evenson 2020 |
|  | ECG (Not Specified) | — | HR accuracy decreased in participants with darker skin | Koerber 2022 |
| **Garmin Vivosmart HR+** | 12-Lead ECG | Low | Vivosmart HR values differed from the ECG HR values for 10 of the 12 resistance exercises, underestimating heart rate during all 12 of them | Evenson 2020 |
|  | Fitbit (Series Unspecified) | High reliability | Good reliability during all tasks, narrower limits of agreement than Fitbit | Fuller 2020 |
| **Garmin  Vivosmart 3** | ECG (Not specified) | — | No HR discrepancies across skin tone | Koerber  2022 |
| **Polar H10** | ECG (Not Specified) | High | 99.67% accuracy, “best model accuracy” | Moshawrab 2023 |
| **Polar OH1** | *** | — | HR accuracy decreased in participants with darker skin | Koerber 2022 |
| **Samsung Gear S** | ECG (Not Specified) | — | No HR discrepancies across skin tone | Koerber 2022 |
| **Samsung Gear S2** | ECG (Not Specified) | — | HR accuracy decreased in participants with darker skin | Koerber 2022 |
|  | Onyx Vantage 9590 clinical pulse oximeter | Low | Accuracies ranged between 80% and 99%, Samsung Gear 2 had the poorest accuracy | Wright 2017 |
| **Samsung Active** | ECG (Not Specified) | High | Samsung Active has significant efficiency in capturing ECG signals, complementing the accuracy of ECGs performed in a doctor’s office, clinic, or hospital room. | Moshawrab 2023 |
| **Mio Alpha** | ECG (Not Specified) | — | One study indicated decreased accuracy with darker skin tones, another one did not | Koerber 2022 |
| **Mio Alpha 2** | ECG (Not Specified) | — | HR accuracy decreased in participants with darker skin | Koerber 2022 |
| **PulseOn** | ECG (Not Specified) | — | HR accuracy decreased in participants with darker skin | Koerber 2022 |
| **Omron HR 500** | ECG (Not Specified) | — | No additional detail | Koerber 2022 |
| **Xiaomi Mi Band (Series Unspecified)** | ECG (Not Specified) | — | No HR discrepancies across skin tone | Koerber 2022 |
| **Basis Peak** | Zephyr Bioharness 3 chest belt | High | Average difference of 0.89 bpm between the two devices | Reeder 2016 |
|  | 12-lead electrocardiograph system | Medium | Accuracies were reduced with increasing exercise intensity for both commercial activity monitors; however, the Fitbit charge was less accurate than the Basis Peak at high-intensity levels, presumably because of factors (e.g., ambient light, sweat, skin contact force) that interfere with the biosensor | Wright 2017 |
| **Health Patch MD** | *** | High | No additional detail | Alharbi 2019 |
| **Jawbone Up** | Onyx Vantage 9590 clinical pulse oximeter | Low precision | Accuracies ranged between 80% and 99%, and the precision ranged between 4% and 18%; Jawbone Up had the poorest precision | Wright 2017 |
| **Misfit (Series Unspecified)** | ECG (Not Specified) | — | No HR discrepancies across skin tone | Koerber 2022 |
| **Misfit Shine** | Onyx Vantage 9590 clinical pulse oximeter | High | MisFit Shine exhibited the highest accuracy and precision | Wright 2017 |

*Note.* *** indicates that information was not reported by the authors. – indicates that some information was reported, but insufficiently to determine a rating.
