# Supplementary material for: Can consumer wearables support outpatient health monitoring for patients with post-acute infection syndromes? A systematic umbrella review of accuracy, validity, and clinical utility data
Source: PLOS Digit Health. 2026 Jun 8;5(6):e0001124. doi: 10.1371/journal.pdig.0001124 (PMC13245765; doi:10.1371/journal.pdig.0001124)
Supplement: S6 Appendix — Note. *** indicates that information was not reported by the authors. – indicates that some information was reported, but insufficiently to determine a rating. (DOCX) [file pdig.0001124.s006.docx]

**S6 Appendix. Atrial Fibrillation Detection accuracy benchmarking**

| **Device** | **Benchmarking Device** | **Overall Conclusions (Low, Medium, or High Accuracy)** | **Additional Detail** | **Article (Year)** |
| --- | --- | --- | --- | --- |
| **Apple Watch (Series Unspecified)** | 12-Lead ECG | High | Sensitivity: 97.92%, Specificity: 99.61% | Belani 2021 |
|  | 12-Lead ECG, Ambulatory ECG post-cardioversion, 7-day ECG patch - single lead | Medium - high | Compared efficacy of Apple Watch with 12-lead ECG and demonstrated sensitivity and specificity of Apple Watch for detecting AF are 93% and 84%, respectively; Specificity of Apple Watches were 98.%, 90% and 84% | Elbey 2021 |
|  | ECG Patches | Medium | No additional detail— | Lui 2022 |
|  | Photoplethysmogram | High | 94.16% accuracy | Moshawrab 2023 |
|  | 12-Lead ECG, Insertable cardiac monitor, ECG patch | — | No additional detail— | Nazarian 2021 |
| **Apple Watch Series 4** | ***– | Low | Apple Watch 4 notification correctly identified AF in 34 of 90 instances (41% sensitivity), with no false positives and 31% inconclusive | Lui 2022 |
| **Apple Watch with KardiaBand** | 12-Lead ECG, Insertable Cardiac Monitor (ICM) | Medium | Sensitivity: 93.0% Specificity: 84.0% Sensitivity: 97.5% Specificity: N/A PPV= 39.9% Sensitivity: N/A Specificity: N/A PPV = 84.0% Sensitivity: 98.4% Specificity: 81.9% PPV = 98.4% | Khundaqji 2021 |
| **Huawei Watch (Series Unspecified)** | 12-Lead ECG | High | 0.99 ES | Nazarian 2021 |
| **Huawei Watch GT** | 12-Lead ECG | High | Sensitivity: 100% Specificity: 98.9% Accuracy = 99.1% Sensitivity: 100% Specificity: 98.93% PPV=91.67% | Khundaqji 2021 |
| **Samsung (Series Unspecified)** | iECG from kardiamobile | High | Cardiologist interpretation of iECG from kardiamobile; Sensitivity: 93.67% Specificity: 98.15% | Belani 2021 |
| **Samsung Gear Fit 2** | iPhone ECG | High | 98.2% specificity | Elbey 2021 |
|  | Mobile ECG | High | Sensitivity: 93.7% Specificity: 98.2% Accuracy = 96.1% | Khundaqji 2021 |
|  | ECG patch | High | 0.96 ES | Nazarian 2021 |
| **Samsung Galaxy Active 2** | Photoplethysmogram | High | Accuracy 91.6%, Specificity 93.0%, Sensitivity 90.8% | Moshawrab 2023 |
| **Samsung Simband** | Single-channel ECG, multi-wavelength photoplethysmography, triaxial accelerometry | High | Specificity was 95%, 97%, and 94% | Elbey 2021 |
|  | Electrocardiogram Photoplethysmogram | High | Sensitivity: 97%, Specificity: 94%, AUROC: 99%; Accuracy 91.8% | Moshawrab 2023 |
| **Moto 360** | Clinical | High | Accuracy > 98.6% | Moshawrab 2023 |
| **Amazfit 1S** | Clinical | High | Sensitivity: 80.00% Specificity: 96.81% Accuracy: 90.52% | Moshawrab 2023 |
|  | 12-Lead ECG, photoplethysmography | High | Accuracy PPG = 93.27%  Accuracy ECG = 94.76% | Khundaqji 2021 |
| **Suunto Movesense** | ECG | High | Accuracy 97.8% | Moshawrab 2023 |
| **KardiaBand** | 12-Lead ECG | Medium - high | Sensitivity: 96.94%, specificity: 81.13% | Belani 2021 |
| **Fitbit Charge HR** | Apple Watch Series 3 | Low | Precision and accuracy for AF detection was higher in Apple Watch Series 3 (75% correlation) as compared to Fitbit (FBT) Charge HR Wireless Activity Wristband (30% correlation) | Elbey 2021 |
| **Honor Band 4** | ECG or 24h ECG Holter | High | 99.2% accuracy, 99.15% specificity | Khundaqji 2021 |
|  | 12-Lead ECG | High | 0.99 ES | Nazarian 2021 |
| **Honor Watch** | ECG or 24h ECG Holter | High | 99.2% accuracy | Khundaqji 2021 |
|  | 12-Lead ECG | High | 0.99 ES | Nazarian 2021 |
| **Non-Specific Device** | Implantable cardiac monitor (ICM) | High | AF-based smartwatches had higher sensitivity for detecting the AF episodes (episode sensitivity 97.5%) and AF duration (duration sensitivity 97.7%) as compared to implantable cardiac monitor | Elbey 2021 |

*Note.* *** indicates that information was not reported by the authors. – indicates that some information was reported, but insufficiently to determine a rating.
