# Supplementary material for: Can consumer wearables support outpatient health monitoring for patients with post-acute infection syndromes? A systematic umbrella review of accuracy, validity, and clinical utility data
Source: PLOS Digit Health. 2026 Jun 8;5(6):e0001124. doi: 10.1371/journal.pdig.0001124 (PMC13245765; doi:10.1371/journal.pdig.0001124)
Supplement: S7 Appendix — Note. *** indicates that information was not reported by the authors. – indicates that some information was reported, but insufficiently to determine a rating. (DOCX) [file pdig.0001124.s007.docx]

**S7 Appendix. Physical Activity: Steps Taken accuracy benchmarking**

| **Device** | **Benchmarking Device** | **Overall Conclusions (Low, Medium, or High Accuracy)** | **Additional Detail** | **Article (Year)** |
| --- | --- | --- | --- | --- |
| **Apple Watch (Series Unspecified)** | Manual count | Medium | The mean difference between the device and the manual count varied from -47 (SD 470) steps to 39.44 (SD 151.81) steps in different walking conditions | Germini 2022 |
|  | *** | High | The walking error was 2.6%; jogging error was 5.1%. Apple Watch displayed a high level of agreement | Lui 2022 |
| **Fitbit (Series Unspecified)** | Direct observation and manual counting | Medium | ~50% of the time, Fitbit devices were likely to provide accurate measures (within ±3%) of steps in controlled testing conditions, with an overall tendency to underestimate steps | Feehan 2018 |
|  | *** | High | High correlation with research grade sensors for step counts. Accuracy varies with placement. FitBit is more accurate at step count when placed at the ankle as compared with the waist | Rao 2019 |
|  | *** | Low | Increases in step counting error rates at slow gait/walking speeds | Strath 2018 |
|  | Research-grade activity monitors | High | Most are as accurate as research-grade activity monitors in counting steps. The error markedly increases at slow walking speeds | Wright 2017 |
| **Fitbit Alta** | Accelerometer | Medium | Mean step count was 773 (SD 829) higher (P=.009) than the one obtained from the reference standard | Germini 2022 |
| **Fitbit Charge** | Automated step counts and direct observation | Medium - High | The mean difference was -59 (SD 704) steps compared with direct observation. The MAPE ranged from -4.4% to 20.7% using different automated step count methods as the reference standard | Germini 2022 |
| **Fitbit Charge 2** | Manual hand counter | High | Accurate and precise for overground walking only | Chevance 2022 |
|  | ActiGraph | Medium | FBC2 overcounted steps; MAPE 12.6% | Irwin 2022 |
| **Fitbit Charge HR** | Video recorded, manual hand counter, | Low - high | Underestimation of steps; accuracy judged as good. Accuracy decreased as walking speed increased; accuracy was judged as not valid for high walking speeds but acceptable for lower walking speeds | Chevance 2022 |
|  | Direct observation or automated step count | Medium | MAPE ranged from –12.7% to 24.1% | Germini 2022 |
|  | ActiGraph and NL2000i | Low | Fitbit Charge HR (wrist) substantially overestimated step count | Straiton 2018 |
| **Fitbit Flex** | Visual step count and GENEactive accelerometer | High | No additional detail | Alharbi 2019 |
|  | Video recording | Medium - high | Pearson CC 0.77-0.85 and mean difference −26.3 to −2.9 | Evenson 2015 |
|  | Manual count or ActiGraph device | Medium - high | Mean percentage error ranged from -23% to 13% | Germini 2022 |
|  | ActiGraph | Medium | Significant correlation Fitbit Flex vs ActiGraph in males, females, total participants and cardiac patients for step counts. Fitbit Flex overestimated step counts in females (556 steps/ day), males (1462 steps/day) and total participants (1038 steps/day) | Straiton 2018 |
| **Fitbit One** | Visual step count | High | Fitbit accurately captured steps at slow speeds when placed at the ankle | Alharbi 2019 |
|  | Video recording | High | Pearson CC 0.99 | Evenson 2015 |
|  | Direct observation | Low | No statistically significant correlation was found in step counting | Germini 2022 |
|  | Visual count | High | Compared to a visual count, the percentage error varied depending on placement of the Fitbit One. At ankle level, agreement was < 10% at speeds of 0.4–0.9 m/s and at waist < 10% for only the 2 fastest speeds 0.8 and 0.9 m/s. Fitbit One accurately captures steps at slow speeds when placed at the ankle. | Straiton 2018 |
|  | *** | Low - high | The error rate was 1% for Fitbit One for normal walking. Fitbit One recorded zero steps for participants walking at 0.3– 0.5 m/s | Wright 2017 |
| **Fitbit Surge** | Manual hand counter | Low | Accurate estimation for the Fitbit Surge at higher walking speeds and inaccurate estimations at lower speeds; Underestimation of steps for all activities, with walking activities being higher than the running | Chevance 2022 |
|  | Direct observation | High | Mean difference compared with direct observation was –86.0 steps (*P=*.004) | Germini 2022 |
| **Fitbit Ultra** | Actical accelerometer and ActiGraph | High | Fitbit Ultra vs. Actical ICC: average 0.94, range 0.80–0.99 (steps). Pearson CC: slow walk: 0.97 (steps: mean 105.3 ActiGraph vs. 105.9 Ultra) | Evenson 2015 |
|  | Manual count | Low | The MAPE was 99.6% (SD 0.8%) and the Pearson correlation coefficient against manual count ranged from 0.44 to 0.99 in different exercise conditions | Germini 2022 |
| **Fitbit Zip** | ActiGraph and Visual count | High | Strong agreement between Fitbit and ActiGraph counted steps | Alharbi 2019 |
|  | Video recording | High | Pearson CC 0.99 | Evenson 2015 |
|  | Direct observation | Low | No statistically significant correlation was found in step counting | Germini 2022 |
|  | Direct observation | Low | All monitors underestimated step counts and energy expenditure (-13% to -32%) and time spent active (-35% to -65%) | Maddocks 2018 |
|  | ActiGraph, Visually counted | High | Good agreement between Fitbit & ActiGraph (ICC2,1 = 0.66, 95% CI 0.41–0.82). Excellent agreement between Fitbit & ActiGraph in average steps/day over 7 days ICC2,1 = 0.94, 95% CI 0.88–0.97). Excellent agreement between Fitbit & visually counted steps (intraclass correlation coefficient (ICC2,1) = 0.88, 95% CI 0.76–0.94) on 2MWT | Straiton 2018 |
| **Fitbit Classic** | Actical | High | Fitbit vs. Actical ICC: average 0.93 | Evenson 2015 |
| **Garmin (Series Unspecified)** | Video observation, gait measurement and analysis device, hand-tally of steps, a pedometer, and an accelerometer | Medium - high | Garmin activity trackers  assessed steps appropriately in most cases. However, there were studies indicating exceptions to this  between 3.1 to 4.0 mph | Evenson 2020 |
| **Garmin Vivofit** | *** | Low - medium | MAPE ranging from –41% to 18% | Germini 2022 |
|  | Video observation | Medium - high | Agreement between the Garmin  activity trackers and walking or running on the treadmill, was good to excellent for the vivofit; Vivofit underestimated steps when walking on flat ground and upstairs, but  overestimated walking downstairs; MAPE was acceptable  (<5%) for slower but not faster speeds on the track | Evenson 2020 |
| **Garmin Vivofit 2** | *** | High | MAPE of 4% | Germini 2022 |
| **Garmin Forerunner 920XT** | Automated step count | High | Different exercise types and conditions, ranged from –2.7% to 1.5% for the Garmin Forerunner 920XT | Germini 2022 |
|  | Video Observation | High | Agreement, between the Garmin  activity trackers and walking or running on the treadmill, was good to excellent for the  Forerunner 920XT | Evenson 2020 |
| **Garmin Vivoactive** | Automated step count | High | MAPE ranged from –1.5% to 0.6% | Germini 2022 |
|  | Video observation | High | Agreement, as indicated by CC between the Garmin  activity trackers and walking or running on the treadmill, was good to excellent for the  Vivoactive | Evenson 2020 |
| **Garmin Vivosmart** | Automated step count | High | MAPE ranged from –1.1% to –0.3% | Germini 2022 |
|  | Hand-tally of steps | High | Agreement, as indicated by CC between the Garmin  activity trackers and walking or running on the treadmill, was good to excellent for the Vivosmart | Evenson 2020 |
| **Garmin Vivoactive HR** | Manual step count | High | Mean difference against manual step count was –19.7 steps (*P=*.03) | Germini 2022 |
| **Garmin Vivosmart HR** | Hand-tally of steps | Low | The CC were lower with faster speed  only for the Vivofit and Vivosmart HR; The Vivosmart HR step counts were not correlated with hand  counted step counts at faster treadmill speeds | Evenson 2020 |
|  | Manual step count | High | Mean difference ranged from –39.7 (SD 54.9) steps to 5.4 (SD 5.8) steps for different walking speeds and locations (outdoor vs indoor) over a total of 111-686 steps | Germini 2022 |
| **Polar V800** | *** | Medium | The Bland–Altman bias was equal to 2487 (SD 2293) steps per day over a mean 10,832 (SD 4578) steps per day | Gemini 2022 |
| **Polar A300** | Bodymedia SWA | High | Polar tracker equivalent to SWA for assessment of PA time, step count and calorie consumption in COPD patients | Alharbi 2019 |
|  | *** | Low - high | Four studies reported an overestimation in daily step counts in five of the eight consumer-grade activity trackers Polar A300) with discrepancies ranging from 167.6 to 2,690.3 steps per day | Straiton 2018 |
|  | *** | High | Pearson correlation coefficient of 0.96 (*P*<.01) | Germini 2022 |
| **Polar Loop** | *** | Low - medium | MAPE for the Polar Loop ranged from –13% to 27% in 3 studies | Germini 2022 |
| **Jawbone (Series Unspecified)** | *** | High | Criterion-referenced validity was high (mean correlations 0.80) compared with that in research- grade monitors for both in-laboratory and free-living scenarios | Strath 2018 |
|  | Research-grade activity monitors | Medium - high | Most are as accurate as research-grade activity monitors in counting steps; however, the error increases at slow walking speeds | Wright 2017 |
| **Jawbone UP** | Bodymedia SenseWear | High | Pearson CC 0.97 | Evenson 2015 |
|  | *** | Medium | The MAPE was –6.73% in one study and the mean absolute difference 806 over an average of 9959 steps in another study | Germini 2022 |
|  | StepWatch | High | Compared the Stepwatch  with four consumer-grade activity trackers worn at the same time, and reported that three (Omron HJ-112, Fitbit One, and Jawbone UP) were accurate at measuring steps, in both non-impaired and impaired ambulation, older adults | Straiton 2018 |
| **Jawbone UP2** | *** | High | The mean difference ranged from 16.19 (SD 29.14) steps to 64 (SD 66.32) steps for different walking conditions over a maximum distance of 1.6 km | Germini 2022 |
| **Jawbone UP24** | *** | High | The mean percentage error ranged from –28% to –0.8% | Germini 2022 |
|  | Direct observation | Low | All monitors underestimated step counts and energy expenditure (-13% to -32%) | Maddocks 2018 |
| **Misfit Shine** | ActiGraph GT3X+ | High | Compared to the ActiGraph GT3X+, the waist-worn Misfit Shine had highest agreement | Alharbi 2019 |
|  | *** | Medium | MAPE ranged from –13% to 23% | Germini 2022 |
|  | ActiGraph | High | Validity was high versus ActiGraph with ICC for Misfit Shine (0.96) | Straiton 2018 |
| **Mio Fuse** | *** | Medium - high | The MAPE ranged from –5% to –16% at different treadmill speeds | Germini 2022 |
| **Nike FuelBand** | *** | Low | The mean percentage error ranged from –34.3% (SD 26.8%) to –16.7% (SD 16.5%) | Germini 2022 |
| **Nike FuelBand SE** | *** | Low - medium | MAPE ranged from 10.2% to 45.0% | Germini 2022 |
| **Omron HJ-112** | StepWatch | High | Compared the Stepwatch  with four consumer-grade activity trackers worn at the same time, and reported that three (Omron HJ-112, Fitbit One, and Jawbone UP) were accurate at measuring steps, in both non-impaired and impaired ambulation, older adults | Straiton 2018 |
| **Omron HJ113-E** | Video Recording | Low | Steps virtually undetected at walking speed of < 0.5 m/s; steps undercounted at w/s> 0.5 m/s | McCullagh 2016 |
| **Omron HJ-720ITC** | Direct Observation | Medium | Accurate at speeds greater than 0.64 m/sec | McCullagh 2016 |
| **Withings Pulse** | *** | High | The MAPE for step count ranged from –16.0% to –0.4% and the accuracy from 97.2% to 99.9% | Germini 2022 |
| **Yamax DW-200** | Direct Observation | Low - high | -25% error (0.95 m/s) to -7% error (1.61 m/s) | McCullagh 2016 |
| **Yamax SW-200** | Direct Observation | Medium - high | Showed good step-count accuracy in walking speeds greater than approximately 1.0 m/sec. -6.8% error in healthy adults; -11.1% error in PD Accuracy decreased with trajectories <5m | McCullagh 2016 |
| **Yamax PW610** | Direct Observation | High | Showed good step-count accuracy in walking speeds greater than approximately 1.0 m/sec | McCullagh 2016 |
| **Digiwalker SW701** | Video Recordings | Medium - high | Lost accuracy below walking speeds of 1.33 m/sec. Pedometer accurately measured step-count | McCullagh 2016 |
| **ADAMO Care Watch** | Manual tally counter | High | DAMO Care Watch demonstrated highly accurate measurements of steps count in all activities, particularly walking at normal and slow speeds | Alharbi 2019 |
| **Kenz Lifecorder** | Direct Observation | Low - high | Mean error step-count, -64.5 to -3.2, "increasingly accurate as walking speed increased." Lost accuracy below walking speeds of 1.33 m/sec. | McCullagh 2016 |

*Note.* *** indicates that information was not reported by the authors. – indicates that some information was reported, but insufficiently to determine a rating.
