# Supplementary material for: Can consumer wearables support outpatient health monitoring for patients with post-acute infection syndromes? A systematic umbrella review of accuracy, validity, and clinical utility data
Source: PLOS Digit Health. 2026 Jun 8;5(6):e0001124. doi: 10.1371/journal.pdig.0001124 (PMC13245765; doi:10.1371/journal.pdig.0001124)
Supplement: S8 Appendix — Note. *** indicates that information was not reported by the authors. – indicates that some information was reported, but insufficiently to determine a rating. (DOCX) [file pdig.0001124.s008.docx]

**S8 Appendix. Energy Expenditure accuracy benchmarking**

| **Device** | **Benchmarking Device** | **Overall Conclusions (Low, Medium, or High Accuracy)** | **Additional Detail** | **Article (Year)** |
| --- | --- | --- | --- | --- |
| **Accelerometers** | “Gold standard doubly labeled water technique” | Low | No additional detail | Shei 2022 |
| **Apple Watch (Series Unspecified)** | Indirect Calorimetry | Low | MAPE ranged from 15% (SD 10%) to 211% (SD ~96%) | Germini 2022 |
|  | Indirect Calorimetry | Medium | Apple Watch showed overestimation of EE. 11% (2/19) of the studies noted an underestimation of total EE in the study group | Lui 2022 |
|  | MAPE extracted from systematic review | Low | Statistically significant differences in average MAPE between age, gender, and BMI categories | Musa 2023 |
|  | Direct or Indirect Calorimetry | Low - medium | Apple wearables overestimated energy expenditure 58% (18/31) of the time | Fuller 2020 |
| **Body Media Fit** | ActiGraph GT3X+ | Medium - high | BodyMedia Fit had absolute error values for energy expenditure similar to ActiGraph GT3X+ (12.6%). BodyMedia was within 10% equivalence of the indirect calorimetry estimate | Wright 2017 |
| **Fitbit (Series Unspecified)** | Direct or Indirect Calorimetry | High | Fitbit overestimated EE during activity (estimated  mean error of 4%). Across comparisons at rest, 3 were within a ±3% measurement error, with 6 lower than –3% and 1 higher than 3%, with a tendency to underestimate energy expenditure (estimated mean error of –3%). | Feehan 2018 |
|  | Direct and Indirect Calorimetry | Low | Fitbit devices underestimated 48.4% of the time and overestimated 39.5% of the time. | Fuller 2020 |
| **Fitbit Alta** | Portable metabolic system -  indirect calorimetry | Medium | Validity coefficient r = 0.63 | Leung 2022 |
| **Fitbit Alta HR** | Portable metabolic system -  indirect calorimetry | Low - medium | Validity coefficient r = 0.56 | Leung 2022 |
| **Fitbit Blaze** | Portable metabolic system -  indirect calorimetry | Low - medium | Validity coefficient = 0.47 | Leung 2022 |
| **Fitbit Charge** | Indirect Calorimetry | Low | MAPE ranged from –4.5% to 75.0% | Germini 2022 |
|  | Portable metabolic system -  indirect calorimetry | Low - medium | Validity coefficient r = 0.53 | Leung 2022 |
| **Fitbit Charge 2** | Indirect Calorimetry | Low | Accuracy judged as poor with significant underestimations | Chevance 2022 |
|  | Portable metabolic system -  indirect calorimetry | Low - medium | Validity coefficient r = 0.50 | Leung 2022 |
| **Fitbit Charge HR** | Indirect Calorimetry (Metamax 3B, CORTEX Bio- physik GmbH) | Medium | Variable during different types of physical activities | Chevance 2022 |
|  | Indirect Calorimetry | Low - medium | MAPE range from –12% to 89% | Germini 2022 |
|  | Portable metabolic system -  indirect calorimetry | Low - medium | Validity coefficient r = 0.55 | Leung 2022 |
| **Fitbit Charge HR 2** | Portable metabolic system -  indirect calorimetry | Low | Validity coefficient = 0.43 | Leung 2022 |
| **Fitbit Classic** | Indirect calorimetry, direct calorimetry, accelerometry (ActiGraph GT3X+), BodyMedia SenseWear, and self-reported data from questionnaire | Low | Regardless of the criterion type, energy expenditure was underestimated | Evenson 2015 |
|  | Portable metabolic system -  indirect calorimetry | Medium | Validity coefficient r = 0.65 | Leung 2022 |
| **Fitbit Flex** | Indirect calorimetry, direct calorimetry, accelerometry (ActiGraph GT3X+), BodyMedia SenseWear, and self-reported data from questionnaire | Low | Regardless of the criterion type, energy expenditure was underestimated | Evenson 2015 |
|  | Indirect Calorimetry | Medium | MAPE of –13% | Germini 2022 |
|  | Portable metabolic system -  indirect calorimetry | Medium | Validity coefficient r = 0.60 | Leung 2022 |
|  | Direct Observation | Low - medium | Underestimation of -13% to -32% | Maddocks 2018 |
| **Fitbit Flex 2** | Portable metabolic system -  indirect calorimetry | Medium | Validity coefficient r = 0.61 | Leung 2022 |
| **Fitbit One** | Indirect calorimetry, direct calorimetry, accelerometry (ActiGraph GT3X+), BodyMedia SenseWear, and self-reported data from questionnaire | Low | Regardless of the criterion type, energy expenditure was underestimated | Evenson 2015 |
|  | Indirect Calorimetry | Medium | Mean bias reports of 2.91 (SD 4.35) kcal per minute | Germini 2022 |
|  | Portable metabolic system -  indirect calorimetry | Medium | Validity coefficient r = 0.62 | Leung 2022 |
|  | Direct Observation | Low - medium | Underestimation of -13% to -32% | Maddocks 2018 |
| **Fitbit Surge** | Indirect calorimetry (Oxycon Mobile) | Low | Underestimation deemed as insufficiently accurate in several studies | Chevance 2022 |
|  | Portable metabolic system -  indirect calorimetry | Medium | Validity coefficient r = 0.64 | Leung 2022 |
| **Fitbit Ultra** | Indirect calorimetry, direct calorimetry, accelerometry (ActiGraph GT3X+), BodyMedia SenseWear, and self-reported data from questionnaire | Low | Regardless of the criterion type, energy expenditure was underestimated | Evenson 2015 |
|  | Indirect Calorimetry | Low | Pearson correlation coefficient ranged from 0.24 to 0.67 for different physical activities | Germini 2022 |
|  | Portable metabolic system -  indirect calorimetry | Medium | Validity coefficient r = 0.75 | Leung 2022 |
| **Fitbit Versa** | Indirect calorimetry (Metamax 3B, CORTEX Bio- physik GmbH) & Jaeger Oxycon Pro | Low - medium | Overestimation of energy expenditure | Chevance 2022 |
| **Fitbit Zip** | Indirect calorimetry, direct calorimetry, accelerometry (ActiGraph GT3X+), BodyMedia SenseWear, and self-reported data from questionnaire | Low | Regardless of the criterion type, energy expenditure was underestimated | Evenson 2015 |
|  | Portable metabolic system -  indirect calorimetry | Low - medium | Validity coefficient r = 0.48 | Leung 2022 |
|  | Direct Observation | Low - medium | Underestimation of -13% to -32% | Maddocks 2018 |
|  | ActiGraph GT3X+ | Medium | Similar absolute error values as the ActiGraph (12.6%) | Wright 2017 |
| **Garmin (Series Unspecified)** | *** | Low | MAPE was  unacceptable | Evenson 2020 |
|  | Direct or Indirect Calorimetry | Low | Underestimation 69% (37/51) of the time | Fuller 2020 |
|  | MAPE extracted from systematic review | Medium | Average MAPE ~15% | Musa 2023 |
| **Garmin Forerunner 225** | Indirect Calorimetry | Low | MAPE ranged from from 31% (SD ~26%) to 155% (SD ~164%) | Germini 2022 |
| **Garmin Forerunner 920XT** | Indirect Calorimetry | Low | MAPE ranged from –27% to 49% | Germini 2022 |
| **Garmin Vivoactive** | Indirect Calorimetry | Low - high | MAPE ranged from 5% to 37% | Germini 2022 |
| **Garmin Vivofit** | Indirect Calorimetry | Low | MAPE ranged from –21% to 45% | Germini 2022 |
| **Garmin Vivosmart** | Indirect Calorimetry | Low - high | MAPE ranged from –2% to –36% | Germini 2022 |
| **Jawbone UP** | *** | Low | Absolute EE estimates were considered “poor” | Maddocks 2018 |
|  | ActiGraph GT3X+ | Medium | Similar absolute error values as the ActiGraph (12.6%) | Wright 2017 |
| **Jawbone UP24** | Indirect calorimetry, direct calorimetry, accelerometry (ActiGraph GT3X+), BodyMedia SenseWear, and self-reported data from questionnaire | Low | Regardless of the criterion type, energy expenditure was underestimated | Evenson 2015 |
|  | Direct Observation | Low - medium | Underestimation of -13% to -32% | Maddocks 2018 |
| **Nike Fuelband** | Indirect Calorimetry | High | Within 10% equivalence of the indirect calorimetry estimate | Wright 2017 |
| **Philips Health Watch** | Indirect Calorimetry | Medium | Error was 10% | Maddocks 2018 |
| **Polar (Series Unspecified)** | Direct or Indirect Calorimetry | Low | Overestimated energy expenditure 69% of the time | Fuller 2020 |
| **Polar A300** | SWA (sensewear) | High | Calories consumption estimates swerve equivalent to SWA (sensewear) in COPD patients | Alharbi 2019 |
|  | Indirect Calorimetry | Medium | Pearson correlation coefficient was 0.74 | Germini 2022 |
| **Polar Loop** | Indirect Calorimetry | Low - high | MAPE ranged from 6% to 56% | Germini 2022 |
| **Polar V800** | Indirect Calorimetry | Low - medium | MAPE ranged from 10% to 40% with a Bland–Altman bias of 957.5 (SD 679.9) kcal. The mean EE measured with the reference standard was 1456.48 (SD 731.40) kcal | Germini 2022 |
| **Wearable Physical Activity Monitors (WPAMs)** | Indirect Calorimetry | Low - medium | Error ranges for EE ranged from  10%–32% | Strath 2018 |
| **Withings Pulse** | Direct or Indirect Calorimetry | Low | Underestimation 74% (34/46) of the time | Fuller 2020 |
|  | Indirect Calorimetry | Low | MAPE ranged from –39% to 64% | Germini 2022 |
| **Xiaomi** | MAPE extracted from systematic review | High | <10 MAPE values | Musa 2023 |

*Note.* *** indicates that information was not reported by the authors. – indicates that some information was reported, but insufficiently to determine a rating.
