# Supplementary material for: Can consumer wearables support outpatient health monitoring for patients with post-acute infection syndromes? A systematic umbrella review of accuracy, validity, and clinical utility data
Source: PLOS Digit Health. 2026 Jun 8;5(6):e0001124. doi: 10.1371/journal.pdig.0001124 (PMC13245765; doi:10.1371/journal.pdig.0001124)
Supplement: S9 Appendix — Note. *** indicates that information was not reported by the authors. – indicates that some information was reported, but insufficiently to determine a rating. (DOCX) [file pdig.0001124.s009.docx]

**S9 Appendix. Total Sleep Time (TST) accuracy benchmarking**

| **Device** | **Benchmarking Device** | **Overall Conclusions (Low, Medium, or High Accuracy)** | **Additional Detail** | **Article (Year)** |
| --- | --- | --- | --- | --- |
| **Fitbit Charge HR** | PSG | Medium | Overestimated TST (8 minutes); sensitivity, 0.97 (SD 0.02); specificity, 0.42 (SD 0.16); Overestimated TST (30 min) | Haghayegh 2019 |
|  | Actigraphy | Medium | No systematic differences between Fitbit and actigraphy in measuring TST; Some overestimation TST (22 min) | Haghayegh 2019 |
|  | Sleep Log | Medium | Fitbit correlated with sleep log (r = .71) for measuring TST; Fitbit Charge HR (r=.58, MAPE=11.5%) | Haghayegh 2019 |
| **Fitbit Surge** | Type III home PSG | Low - medium | Normal mode overestimated TST (46 min), 0.88 (SD 0.05); Sensitive mode underestimated TST (86 min), accuracy, 0.78 (SD 0.08) | Haghayegh 2019 |
|  | Actigraphy | Low - medium | Normal overestimated TST (5 min); Sensitive mode underestimated TST (127 min) | Haghayegh 2019 |
| **Fitbit Flex** | PSG | Medium - high | Normal mode overestimated TST (46 min); Sensitive mode underestimated TST (86 min); TST by Fitbit and PSG correlated (⍴=.99); average percentage error: 2.97% | Haghayegh 2019 |
|  | Actigraphy | Low - high | Normal mode overestimated TST (5 min; Sensitive mode underestimated TST (127 min) | Haghayegh 2019 |
|  | Sleep Log | High | MAPE =8.80%, r=0.68 correlated with sleep log | Haghayegh 2019 |
| **Fitbit Alta HR** | PSG | High | Overestimated TST (12 min); accuracy, 0.90 (SD 0.04) | Haghayegh 2019 |
|  | Sleep Log | High | Underestimated TST (6 min) | Haghayegh 2019 |
| **Fitbit Charge 2** | PSG | High | Overestimated TST (9 min) | Haghayegh 2019 |
|  | Sleep Scope (EEG based) | High | Underestimated TST (12 min) | Haghayegh 2019 |
| **Fitbit Alta** | Actigraphy | Low - medium | Good sleepers overestimated TST (74 min); Poor sleepers overestimated TST (20 min) | Haghayegh 2019 |
| **Fitbit One** | *** | High | Pearson CC 0.92 | Evenson 2015 |
|  | PSG | Medium | Overestimated TST (88 min); accuracy, 0.81 | Haghayegh 2019 |
| **Fitbit Ultra** | PSG | Low | Overestimated TST resulting in high sensitivity and poor specificity. Under sensitive mode, TST was underestimated;  Normal mode overestimated TST by 41 minutes; Sensitive mode underestimated TST by 105 minutes, 71% accuracy | Evenson 2015 |
|  | PSG | Low - medium | Normal mode overestimated TST by 41 min; Sensitive mode underestimated TST by 105 min | Kolla 2016 |
|  | Actigraphy | Low | Tendency to overestimate TST in normal mode and to underestimate TST in sensitive mode; A substantial number of subjects with discrepancies of >30 min in TST (8% and 42% of subjects compared to two types of actigraphy) | Kolla 2016 |
|  | Pair (wearing two of same device) | High | Similar readings were found for TST for either normal or sensitive mode | Evenson 2015 |
| **Fitbit Classic** | PSG | Low | Overestimated TST by mean 67.1 min (SD 51.3) | Evenson 2015 |
|  | PSG | Low | Overestimated TST      (67 min) | Haghaeygh 2019 |
|  | Actigraphy | Medium | Overestimated TST (24 min) | Haghaeygh 2019 |
|  | Pair wearing two of same device) | High | High levels of agreement (96.5 -- 99.1%) | Evenson 2015 |
| **Fitbit Versa** | Sleep Scope (EEG based) | High | Overestimated TST (7 min); accuracy, 0.89 | Haghayegh 2019 |
| **Fitbit (Series Unspecified)** | PSG | Medium | Normal mode setting reported Fitbit overestimation of TST by more than 10% | Feehan 2018 |
|  | Sensewear accelerometer, Actiwatch accelerometer | High | Measurement errors within ±10%; Overestimate (6%) of sleeping minutes for Fitbit compared with Actiwatch accelerometer | Feehan 2018 |
| **Jawbone** | PSG | Medium | Data from Jawbone differed significantly from PSG for TST (−10 ± 20.5 min); 14.4% of subjects with >30 min discrepancy in TST; overestimated TST (26.6 ± 35.3 min) | Kolla 2016 |
| **Jawbone Up** | PSG | Medium - high | Overestimated TST by 26.6 ± 35.3 min (p < 0.001); Overestimated TST by 10.0 min (p < 0.001) | Evenson 2015 |
|  | PSG | Medium | Significant disagreements between device and PSG were noted in differences in TST (>30 min) in 36% of participants | Kolla 2016 |
|  | Actigraphy | High | TST showed no difference between devices | Kolla 2016 |
| **Vivofit** | Sleep Diary | High | Good CC and acceptable MAPE with mean sleep time similar between measures | Evenson 2020 |
| **Vivosmart** | Sleep Diary | Low | Overestimated mean sleep time; low agreement compared to diary measures; findings were generally poor and variable | Evenson 2020 |

*Note.* *** indicates that information was not reported by the authors. – indicates that some information was reported, but insufficiently to determine a rating.
