# Supplementary material for: Can consumer wearables support outpatient health monitoring for patients with post-acute infection syndromes? A systematic umbrella review of accuracy, validity, and clinical utility data
Source: PLOS Digit Health. 2026 Jun 8;5(6):e0001124. doi: 10.1371/journal.pdig.0001124 (PMC13245765; doi:10.1371/journal.pdig.0001124)
Supplement: S10 Appendix — Note. *** indicates that information was not reported by the authors. – indicates that some information was reported, but insufficiently to determine a rating. (DOCX) [file pdig.0001124.s010.docx]

**S10 Appendix. Sleep Efficiency (SE) accuracy benchmarking**

| **Device** | **Benchmarking Device** | **Overall Conclusions (Low, Medium, or High Accuracy)** | **Additional Detail** | **Article (Year)** |
| --- | --- | --- | --- | --- |
| **Fitbit Charge HR** | PSG | High | Overestimated SE (1.8%) | Haghayegh 2019 |
|  | Actigraphy | High | No additional detail | Haghayegh 2019 |
|  | Sleep Log | Low | No correlation between Fitbit and sleep log | Haghayegh 2019 |
| **Fitbit Surge** | PSG | Medium - high | Normal mode overestimated SE (8.1%); Sensitive mode underestimated SE (16.0%) | Haghayegh 2019 |
|  | Actigraphy | Medium - high | Normal mode overestimated SE (1.1%); Sensitive mode underestimated SE (22.9%) | Haghayegh 2019 |
| **Fitbit Flex** | PSG | Medium - high | Normal mode overestimated SE (8.1%); Sensitive mode underestimated SE (16.0%) | Haghayegh 2019 |
|  | Actigraphy | Medium - high | Normal mode overestimated SE (1.1%); Sensitive mode underestimated SE (22.9%) | Haghayegh 2019 |
| **Fitbit Alta HR** | PSG | High | Overestimation of SE (2.0%) | Haghayegh 2019 |
|  | Sleep Log | High | Overestimation of SE (1.4%) | Haghayegh 2019 |
| **Fitbit Charge 2** | Sleep Scope (EEG based) | High | Underestimation of SE (1.5%) | Haghayegh 2019 |
| **Fitbit One** | PSG | Medium | Overestimation of SE (17.4%) | Haghayegh 2019 |
| **Fitbit Ultra** | PSG | Medium - high | Normal mode underestimated SE (8%); Sensitive mode underestimated SE (21%); accuracy, 0.71 | Haghayegh 2019 |
|  | PSG | Medium - high | Normal mode overestimated SE  (8%) ; Sensitive mode underestimated SE by (21%) | Kolla 2016 |
|  | Actigraphy | Medium - high | Overestimation of SE in normal mode and to underestimate SE sensitive mode | Kolla 2016 |
| **Fitbit Classic** | PSG | Medium | Overestimation of SE (14.5%) | Haghayegh 2019 |
|  | Actigraphy | High | Overestimation of SE (5.2%) | Haghayegh 2019 |
| **Fitbit Versa** | Sleep Scope  (EEG based) | High | Underestimation of SE (0.1%) | Haghayegh 2019 |
| **Fitbit (Series Unspecified)** | PSG | Medium | Normal mode setting overestimated SE by more than 10%; Sensitive mode underestimated SE by more than 15% | Feehan 2018 |
| **Jawbone** | PSG | Medium - high | Overestimation of SE; data differed significantly from PSG (−1.9 ± 4.2%); 26.2% of subjects with a >5% discrepancy in SE | Kolla 2016 |
| **Jawbone Up** | PSG | High | Overestimation of SE by 1.9 % (p < 0.001) | Evenson 2015 |
|  | PSG | Medium - high | Device did not differ significantly on mean measurements of SE; Significant disagreements in SE measurements: (>5%) in 47% of the participants | Kolla 2016 |
|  | Actigraphy | High | No difference in SE data between these two devices | Kolla 2016 |
| **Vivosmart** | Sleep Diary | Low | “Other measures of sleep (SE) were also not well measured.” | Evenson 2020 |

*Note.* *** indicates that information was not reported by the authors. – indicates that some information was reported, but insufficiently to determine a rating.
