# Supplementary material for: Can consumer wearables support outpatient health monitoring for patients with post-acute infection syndromes? A systematic umbrella review of accuracy, validity, and clinical utility data
Source: PLOS Digit Health. 2026 Jun 8;5(6):e0001124. doi: 10.1371/journal.pdig.0001124 (PMC13245765; doi:10.1371/journal.pdig.0001124)
Supplement: S11 Appendix — Note. *** indicates that information was not reported by the authors. – indicates that some information was reported, but insufficiently to determine a rating. + Authors of this article conducted multiple tests of similarities and differences; here, we have extracted and reported just Bland-Altman analyses as a concise indicator of agreement between the two measurement methods. The authors full results can be found in their supplementary materials at: Appendix A: Supplementary Materials. (DOCX) [file pdig.0001124.s011.docx]

**S11 Appendix. Sleep Onset Latency (SOL) accuracy benchmarking**

| **Device** | **Benchmarking Device** | **Overall Conclusions (Low, Medium, or High Accuracy)** | **Additional Detail** | **Article (Year)** |
| --- | --- | --- | --- | --- |
| **Fitbit Charge HR** | PSG | High | Overestimation of SOL (3 min) | Haghayegh 2019 |
| **Fitbit Surge** | Home PSG | Medium - high | Normal mode underestimates SOL (2 min); Sensitive mode overestimates SOL (12 min) | Haghayegh 2019 |
|  | Actigraphy | Medium | Normal mode overestimates SOL (12 min); Sensitive mode overestimates SOL (25 min) | Haghayegh 2019 |
| **Fitbit Flex** | Consumer Actigraphy Device | — | Normal settings were more accurate for estimating SOL than the sensitive setting for individuals with Major Depressive Disorder; Higher ICC for the normal settings compared to the sensitive settings for individuals with insomnia and “good sleepers” | Scott 2020 |
|  | PSG & Unattended PSG | Medium - high | Normal mode underestimated  SOL (2 min) and Sensitive mode overestimated SOL (12 min); Normal mode overestimated SOL (1 min); For insomniacs using normal mode, the device underestimated SOL (2.4%) | Haghayegh 2019 |
|  | Actigraphy | Medium | Normal mode overestimated SOL (12 min); Sensitive mode overestimated SOL (25 min) | Haghayegh 2019 |
| **Fitbit Alta HR** | PSG | High | Underestimation of SOL (4 min) | Haghayegh 2019 |
|  | Sleep Log | High | Overestimation of SOL (5 min) | Haghayegh 2019 |
| **Fitbit Charge 2** | PSG | Low - medium | + “Discrepancy (SD) between PSG and Device, or Bland-Altplan Plot statistics: Main group, Bias = 4 (SD = 9), lower limit: -14, upper limit 23. PLMS group, bias = 7 (SD = 10), lower limit: -14, upper limit: 27 | Scott 2020 |
|  | PSG | High | Normal sleeper cohort underestimated SOL (4 min); periodic limb movement in sleep  (PLMS) cohort underestimated SOL (7 min) | Haghayegh 2019 |
|  | Sleep Scope (EEG based) | Medium | Underestimated SOL  (11 min) | Haghayegh 2019 |
| **Fitbit One** | PSG | Medium | Underestimated SOL (17 min) | Haghayegh 2019 |
| **Fitbit Versa** | Sleep Scope (EEG based) | Medium | Underestimated SOL (14 min) | Haghayegh 2019 |
| **Fitbit (Series  Unspecified)** | *** | Low - medium | Measurement errors varying from 12% to 180% with varying over- or underestimation depending on the sleep setting | Feehan 2018 |
| **Jawbone** | PSG | High | The device did not differ significantly from PSG; Overestimation of SOL (5.2 ± 9.6 min) | Kolla 2016 |
| **Jawbone Up** | *** | Low | + “Bland-Altman Plot statistics: M = 5.2 (SD = 9.6), lower limit: -24.1, upper limit: 13.7;  Correlation between PSG & Device (95% CI): UP: not significant” | Scott 2020 |
|  | Actigraphy | Low | Significantly longer SOL measurements (21 min) | Kolla 2016 |
|  | PSG | High | No significant differences between devices on SOL mean measurements | Kolla 2016 |
|  | PSG | Medium - high | Overestimation of SOL by 5.2 ± 9.6 min (p = 0.005); Overestimation of SOL by 1.3 min (p = 0.33) | Evenson 2015 |
| **Jawbone Up 3** | *** | Medium | +“Discrepancy (SD) between PSG and Device, or Bland-Altman Plot statistics: UP3-PSG: M = -5.13, not significant” | Scott 2020 |
| **Withings Pulse O2** | PSG | Low | “SOL MEANS (SD): PSG: M = 14 (SD = 13)  Withings: M = 13 (SD = 4), non significant;.  Correlation between PSG & Device (95% CI): Withings: non significant” | Scott 2020 |
| **Zeo wireless system** | PSG | Medium | High correspondence between; ICC: 0.42 to 0.67; Significant underestimation in some studies, while others have no significant differences | Scott 2020 |

*Note.* *** indicates that information was not reported by the authors. – indicates that some information was reported, but insufficiently to determine a rating. + Authors of this article conducted multiple tests of similarities and differences; here, we have extracted and reported just Bland-Altman analyses as a concise indicator of agreement between the two measurement methods. The authors full results can be found in their supplementary materials at: [Appendix A: Supplementary Materials](https://ars.els-cdn.com/content/image/1-s2.0-S1087079219301959-mmc1.docx).
