# Supplementary material for: Can consumer wearables support outpatient health monitoring for patients with post-acute infection syndromes? A systematic umbrella review of accuracy, validity, and clinical utility data
Source: PLOS Digit Health. 2026 Jun 8;5(6):e0001124. doi: 10.1371/journal.pdig.0001124 (PMC13245765; doi:10.1371/journal.pdig.0001124)
Supplement: S12 Appendix — Note. *** indicates that information was not reported by the authors. – indicates that some information was reported, but insufficiently to determine a rating. (DOCX) [file pdig.0001124.s012.docx]

**S12 Appendix. Wake After Sleep Onset (WASO) accuracy benchmarking**

| **Device** | **Benchmarking Device** | **Overall Conclusions (Low, Medium, or High Accuracy)** | **Additional Detail** | **Article (Year)** |
| --- | --- | --- | --- | --- |
| **Fitbit Charge HR** | PSG | High | Underestimation of WASO (6 min) | Haghayegh 2019 |
|  | Sleep Log | Low | No correlation of WASO | Haghayegh 2019 |
| **Fitbit Surge** | PSG | Low | Normal mode underestimated WASO (44 min); Sensitive mode overestimated WASO (75 min) | Haghayegh 2019 |
|  | Actigraphy | Low - medium | Normal mode underestimated WASO (17 min); Sensitive mode overestimated WASO (102 min) | Haghayegh 2019 |
| **Fitbit Flex** | PSG & Unattended PSG | Low - medium | Normal mode underestimated WASO (44 min) and Sensitive mode overestimated WASO (75 min) | Haghayegh 2019 |
|  | Actigraphy | Low - medium | Normal mode underestimated WASO (17 min); Sensitive mode overestimated WASO (102 min) | Haghayegh 2019 |
| **Fitbit Alta HR** | PSG | High | Underestimation of WASO (8 min) | Haghayegh 2019 |
|  | Sleep Log | Medium | Overestimation of WASO (13 min) | Haghayegh 2019 |
| **Fitbit Charge 2** | PSG | High | Underestimation of WASO (5 min) for normal sleepers; Periodic limb movement in sleep  (PLMS) cohort underestimated WASO (1 min) | Haghayegh 2019 |
|  | Sleep Scope (EEG based) | Medium | Overestimation of WASO (25 min) | Haghayegh 2019 |
| **Fitbit Alta** | PSG | High | Underestimation of WASO (8 min) | Haghayegh 2019 |
|  | Actigraphy | Medium | Underestimation of WASO for good sleepers (16 min); underestimation of WASO for poor sleepers (13 min) | Haghayegh 2019 |
|  | Sleep Log | Medium | Overestimation of WASO (13 min) | Haghayegh 2019 |
| **Fitbit One** | PSG | Medium | Underestimation of WASO (39 min) | Haghayegh 2019 |
| **Fitbit Ultra** | PSG | Low - medium | Normal mode underestimated WASO (32 min); Sensitive mode overestimated WASO (106 min) | Haghayegh 2019 |
| **Fitbit Versa** | Sleep Scope (EEG based) | Medium | Overestimation of WASO (14 min) | Haghayegh 2019 |
| **Fitbit (Series Unspecified)** | *** | Low - medium | Measurement errors varying from 12% to 180% with varying over- or underestimation depending on the sleep setting | Feehan 2018 |
| **Jawbone** | PSG | Medium | WASO measurements significantly differed from PSG (10.6 ± 14.7 min); 10.8% of subjects with >30 min discrepancy in WASO | Kolla 2016 |
| **Jawbone Up** | PSG | Medium | Underestimation of WASO by 31.2 ± 32.3 min (p < 0.001) | Evenson 2015 |
|  | Actigraphy | Low - medium | WASO measurements were significantly shorter (13 min) from Jawbone measurements | Kolla 2016 |
|  | PSG | Low - Medium | Significant disagreements between devices in measurements of WASO (>30 min) for 41% of participants | Kolla 2016 |

*Note.* *** indicates that information was not reported by the authors. – indicates that some information was reported, but insufficiently to determine a rating.
