# Supplementary material for: Can consumer wearables support outpatient health monitoring for patients with post-acute infection syndromes? A systematic umbrella review of accuracy, validity, and clinical utility data
Source: PLOS Digit Health. 2026 Jun 8;5(6):e0001124. doi: 10.1371/journal.pdig.0001124 (PMC13245765; doi:10.1371/journal.pdig.0001124)
Supplement: S13 Appendix — Note. *** indicates that information was not reported by the authors. – indicates that some information was reported, but insufficiently to determine a rating. (DOCX) [file pdig.0001124.s013.docx]

**S13 Appendix. Other Sensor Data accuracy benchmarking**

| **Device** | **Sensor Data** | **Benchmarking Device** | **Overall Conclusions (Low, Medium, or High Accuracy)** | **Additional Detail** | **Article (Year)** |
| --- | --- | --- | --- | --- | --- |
| Fitbit (Series Unspecified) | Distance | Treadmill distance | Medium - high | Tendency to overestimate distance at slower and self-paced speeds (5% [torso] to 25% [wrist]) and underestimate distance at brisk walking or jogging speeds (–15% [wrist] to –5% [torso]). Torso placement during normal ambulation speed tended to overestimate distance (10%) while wrist placement tended to underestimate distance (–3%) | Feehan 2018 |
| Fitbit Flex | Physical activity duration | Research-grade monitors | — | Overestimation (average 10 min/day) | Straiton 2018 |
|  | Time Spent in Moderate- to Vigorous-Intensity Physical Activity (MVPA) | “3 different devices” | Low - medium | MAPE varied from 7% (SD 6%) to 74% (SD 13%) and mean percentage error ranged from –65% to 10% | Germini 2022 |
| Fitbit One | Distance | Manual Count | Low | Significant differences between observed and Fitbit One distance measurements; ICC 0.0-0.05, relative error 5.0-39.6 % | Evenson 2015 |
| Polar A300 | Physical activity duration | Research-grade monitors | Low | Considered highly variable | Straiton 2018 |
| SmartMonitor Smartwatch | Seizure activity | Video EEG data recordings | Low | ⅞ seizures were detected by the smartwatch. Missed detection was attributed to either uncharged battery or failed Bluetooth connection. A false seizure was detected during a sleep period; SmartWatch detected 16/51   (31%) of generalized tonic-clonic seizures, 3/32 (6%) of myoclonic/myoclonic tonic, and 11/45 (24%) of partial-onset seizures with motor component with an overall detection rate of 16% | Reeder 2016 |
| Apple Watch Series 4 | ECG Signal Quality | 12-lead ECG | High | Apple Watch yielded good diagnostic signal quality and  morphology | Bouzid 2022 |
| HealthPatch MD | Respiratory rate | XPREZZON: ICU grade’ patient monitor | Low | Accurate measurement of HR, but not for respiratory rate | Alharbi 2019 |

*Note.* *** indicates that information was not reported by the authors. – indicates that some information was reported, but insufficiently to determine a rating.
