# Supplementary material for: Can consumer wearables support outpatient health monitoring for patients with post-acute infection syndromes? A systematic umbrella review of accuracy, validity, and clinical utility data
Source: PLOS Digit Health. 2026 Jun 8;5(6):e0001124. doi: 10.1371/journal.pdig.0001124 (PMC13245765; doi:10.1371/journal.pdig.0001124)
Supplement: Attachment — Submitted filename: PASC Review Response to Reviewers.docx [file pdig.0001124.s015.docx]

*Author explanations for how each comment has been responded to appear in italicized text after each point below.*

**Journal Requirements:**

1. Please amend your detailed Financial Disclosure statement. This is published with the article. It must therefore be completed in full sentences and contain the exact wording you wish to be published.

*-Reply: The financial disclosure statement has been revised according to these instructions in the journal portal.*

i. Please clarify all sources of financial support for your study. List the grants, grant numbers, and organizations that funded your study, including funding received from your institution. Please note that suppliers of material support, including research materials, should be recognized in the Acknowledgements section rather than in the Financial Disclosure.
ii. State the initials, alongside each funding source, of each author to receive each grant. For example: "This work was supported by the National Institutes of Health (####### to AM; ###### to CJ) and the National Science Foundation (###### to AM)."
iii. State what role the funders took in the study. If the funders had no role in your study, please state: “The funders had no role in study design, data collection and analysis, decision to publish, or preparation of the manuscript.”
iv. If any authors received a salary from any of your funders, please state which authors and which funders.

*-Reply*: *This information has been added to the financial disclosure statement provided in the journal portal.*

2. Please send a completed 'Competing Interests' statement, including any COIs declared by your co-authors. If you have no competing interests to declare, please state "The authors have declared that no competing interests exist". Otherwise please declare all competing interests beginning with the statement "I have read the journal's policy and the authors of this manuscript have the following competing interests:"

*-Reply: Our Competing Interests statement has been updated in the journal portal according to these guidelines.*

3. Please upload separate figure files in .tif or .eps format. Also, remove the figures from your manuscript file but keep the legends.For more information about figure files please see our guidelines: <https://journals.plos.org/digitalhealth/s/figures>
[https://journals.plos.org/digitalhealth/s/figures#loc-file-requirements](https://nam11.safelinks.protection.outlook.com/?url=https%3A%2F%2Furl.usb.m.mimecastprotect.com%2Fs%2Fz_EuCP6q0kCoZg0wqHjsJIxxerj%3Fdomain%3Djournals.plos.org&data=05%7C02%7Cdeanna.m.kaplan%40emory.edu%7C3cfc305579594bacc51c08de645aac0b%7Ce004fb9cb0a4424fbcd0322606d5df38%7C0%7C0%7C639058537790121705%7CUnknown%7CTWFpbGZsb3d8eyJFbXB0eU1hcGkiOnRydWUsIlYiOiIwLjAuMDAwMCIsIlAiOiJXaW4zMiIsIkFOIjoiTWFpbCIsIldUIjoyfQ%3D%3D%7C0%7C%7C%7C&sdata=1FQRBWEihUkqOlij8y3InNz921HpI6SuN6CQxfQsOUM%3D&reserved=0)

*-Reply: These changes have been made as requested.*

4. We have noticed that you have uploaded Supporting Information files, but you have not included a list of legends. Please add a full list of legends for your Supporting Information files after the references list.

*-Reply: We have added a List of Legends for Supporting Information Files to the main manuscript after the References section, as requested.*

5. As required by our policy on Data Availability, please ensure your manuscript or supplementary information includes the following:

A numbered table of all studies identified in the literature search, including those that were excluded from the analyses.

For every excluded study, the table should list the reason(s) for exclusion.

If any of the included studies are unpublished, include a link (URL) to the primary source or detailed information about how the content can be accessed.

*-Reply: As discussed in the methods section, this review utilized intentionally broad search criteria as this was necessary to address the objectives of our review, and the original literature search included 6,836 titles. 2,848 of these were found to be duplicates, and our team double-screened the remaining 3,988 (see Fig 1, PRISMA diagram). This makes providing a table of all originally identified studies impractical (and indeed, this exceeds the ability of the Covidence review software to generate). Given this, we have instead provided a numbered table of all studies that made it to the stage of full text review (n=100) with reasons for exclusion at this stage. This is now provided on the OSF supplement for the project under the project component “Excluded Studies with Reason” accessible at: https://osf.io/4sxnf/. Detailed information about how the content can be accessed is provided within this table.*

A table of all data extracted from the primary research sources for the systematic review and/or meta-analysis. The table must include the following information for each study:

Name of data extractors and date of data extraction

Confirmation that the study was eligible to be included in the review.

*-Reply: Due to the size of our review it was necessary to provide this information across two tables, but this information is now provided. A table of all data extracted can be found on the OSF supplement for this project under the component “Extraction Template and Extracted Data”. A table that contains the name of data extractors and date of data extraction can now be found in the newly added component “Extractor Names and Dates”. Confirmation that included studies were eligible for inclusion in the review can be found both on the OSF page and in the main manuscript.*

All data extracted from each study for the reported systematic review and/or meta-analysis that would be needed to replicate your analyses.

-*Reply: This can be found in the OSF project component “Extraction Template and Extracted Data”.*

-If data or supporting information were obtained from another source (e.g. correspondence with the author of the original research article), please provide the source of data and dates on which the data/information were obtained by your research group.

*-Reply: This is not applicable to our project.*

If applicable for your analysis, a table showing the completed risk of bias and quality/certainty assessments for each study or outcome. Please ensure this is provided for each domain or parameter assessed. For example, if you used the Cochrane risk-of-bias tool for randomized trials, provide answers to each of the signalling questions for each study. If you used GRADE to assess certainty of evidence, provide judgements about each of the quality of evidence factor. This should be provided for each outcome.

*-Reply: This is provided in the manuscript supplementary materials as Appendix 3.*

An explanation of how missing data were handled.

-*Reply: We have added the following statement about missing data to the revision of our manuscript under the “Data extraction” heading of our methods section: “Missing data (e.g., variables in the extraction template that were not included in a given review) were extracted as “not included.” (p. 10)*

This information can be included in the main text, supplementary information, or relevant data repository. Please note that providing these underlying data is a requirement for publication in this journal, and if these data are not provided your manuscript might be rejected.

If the reviewer comments include a recommendation to cite specific previously published works, please review and evaluate these publications to determine whether they are relevant and should be cited. There is no requirement to cite these works unless the editor has indicated otherwise.

*-Reply: We have included one new citation provided to us by Reviewer 1. Reviewer 1 mentioned additional appended references in their comment #7, but unfortunately these were not appended to their reviewer comments. We are happy to consider additional citations although we also note that the citation list for this article is already long at 95 references.*

**Additional Editor Comments (if provided):**

Dear Dr. Kaplan and co-authors,

We thank you for submitting your manuscript to *PLOS Digital Health* and for your thorough and timely work on this highly relevant topic. This pre-registered systematic umbrella review provides a valuable synthesis of evidence on the accuracy, validity, and clinical utility of consumer wearables for biometrics relevant to post-acute infection syndromes, including Long COVID (PASC). The rigorous methodology—adherence to PRISMA guidelines, PROSPERO registration, AMSTAR 2.0 quality appraisal, double data extraction, and full data availability on OSF—is exemplary. The inclusion of 42 reviews covering more than 30 brands and over 150 device series offers comprehensive coverage, and the identification of stronger evidence for heart rate measurement and atrial fibrillation detection, alongside limited support for other biometrics, is clearly presented. The supplementary device-level benchmarking tables and especially the innovative living review repository on the Open Science Framework are significant strengths that enhance the practical utility and longevity of this work for researchers, clinicians, and patients. Both reviewers found the manuscript technically sound, well-written, and important, but identified opportunities to strengthen its impact. To better serve our interdisciplinary readership, we ask you to revise the manuscript.

*-Reply: We appreciate the editors’ encouraging words about our work, as well as the excellent suggestions that follow. We have incorporated all suggestions provided and feel that these additions have improved the quality of this contribution.*

(1) provide clearer clinical translation of the findings into practical guidance for self-monitoring, activity pacing, screening, and clinician-supported use in PASC and related syndromes, explicitly distinguishing direct evidence from PASC populations versus extrapolation from other groups;

*-Reply: To address this, we have made considerable revisions to the final subsection of the Discussion (“Are we there yet? Conclusions and recommendations about consumer wearables for outpatient health monitoring” found on pp. 40-42) to focus on clinical translation and recommendations. As part of this, we have also introduced a new figure (Fig 8) that provides 5 specific recommendations for patients who are already using a wearable or want to use one, guided by findings from our review.*

*To address the final point about explicitly distinguishing direct evidence from extrapolation, we have added the following text to the limitations section of the manuscript: ““As noted previously, this umbrella review employed broad search and inclusion criteria given that PASC only emerged as a recognized diagnosis in 2020 and limited data for individuals with others post-acute viral syndromes exist. Reflecting this paucity of data and the broader tendency for wearable research to focus on healthy populations, only 7 of 42 reviews examined populations with a clinical or morbidity characteristics at all. Accordingly, this review provides extrapolated findings with relevance to PASC and post-acute infection syndromes and should not be interpreted as direct evidence.” (p. 39)*

(2) improve synthesis and presentation of the heterogeneous evidence through additional summaries, tables, or visual aids that highlight consistent patterns and facilitate interpretation;

*-Reply: We thought this was an excellent suggestion and carefully weighed how to best provide a concise visual aid that highlights consistent patterns without inadvertently masking the heterogeneity of the evidence. To accomplish this, we have created a new figure (Fig 4 of this revision) that provides a heat map of (1) the volume of evidence for each biometric, and (2) the overall evaluation of accuracy based on that evidence, alongside (3) a brief summary with any major cautions noted.*

(3) expand discussion of equity considerations, including demographic gaps in validation studies (e.g., skin tone, race/ethnicity, age, socioeconomic status), differential accuracy, and implications for safety and access;

*-Reply: To address this, we have expanded a subsection of the Discussion (now titled: “Equity and underrepresentation: Do wearables work equally well for everyone?”) To this section, we have added the following new text: “This study identified a concerning lack of review-level evaluation of device performance across diverse demographic groups. While age (reported in 74.5% of reviews) and gender (reported in 69% of reviews) were frequently addressed, race and ethnicity were rarely examined, appearing in only 7% (k=3) reviews and fewer than 5% (k=2) reviews, respectively. When demographic factors were examined, findings often suggested diminished performance for some biometrics and devices based on these characteristics—for example, the finding that photoplethysmography (PPG) sensors (used commonly for HR measurement in wristwatch devices) may be less accurate in individuals with darker skin tones or more body hair. Socioeconomic status was also infrequently included (14% or k=6 reviews), despite its clear relevance to wearable device access. Many wearable devices depend on smartphone pairing and reliability internet connectivity, which may systematically exclude individuals with lower incomes or those living in rural areas. These equity issues are highly intersectional, given that individuals from lower income backgrounds are disproportionately represented among historically underrepresented racial and ethnic groups. Together, these gaps raise concerns about the generalizability and equity of the current evidence, and highlight a need for more inclusive primary studies and consistent reporting of demographic characteristics in future research.” (pp. 35-36)*

(4) deepen analysis of provider acceptability (e.g., EHR integration challenges, information overload, algorithmic transparency), potential adverse events or harms in PASC patients (e.g., anxiety from false alerts, compulsive checking), and reasons for gaps in reporting these outcomes;

*-Reply: We have addressed these points following the excellent suggestions of the reviewers of this article.*

*To deepen our analysis of provider acceptability, we have added the following new text to the text: “Other gaps in the literature included limited data about acceptability from the perspective of providers, which may represent a significant barrier to implementation. Health information technology-related stress is a well-established predictor of physician burnout (75,76). Although this body of research has primarily focused on electronic health record (EHR) systems rather than adjunctive technologies such as wearables, it foreshadows broader implementation challenges that warrant targeted research. Potential barriers to provider acceptability include increased workload associated with reviewing additional streams of patient health data, challenges integrating wearable-assessed data into EHR systems, and concerns related to the privacy and security of these health data.” (p. 33)*

*To deepen our analysis of potential adverse events or harms and reasons for gaps in reporting these outcomes, we have added the following text to our Discussion: “This review was unable to identify any review-level reporting of adverse events or adverse effects at all, including an absence of evidence for these effects. Several constraints hamper the assessment of adverse effects in consumer wearables research. Adverse effects assessment in studies of wearable technologies has never been systematized, and no articles in this review describe any formal method for AE assessment. This suggests that, as in other research areas (78,79), studies typically rely on passive monitoring (i.e., adverse events are only recorded if a participant volunteers one), rather than specific solicitation. And yet, passive monitoring under-counts AEs by up to a factor of 20 (80) compared to AE solicitation. For FDA-regulated research, which includes the investigation of medical devices, current AE assessment frameworks rely on medical and pharmacological lexica such as the Medical Dictionary for Regulatory Activities (MEDdra; 83) or Common Terminology Criteria for Adverse Events (CTCAE; 84). However, reliance on such criteria are known to systematically under-count AEs in behavioral and lifestyle interventions (83), suggesting that these lexica may not be sufficient for research on consumer wearables.” (pp. 33-34)*

*To address harms specifically relevant to PASC patients, we have added the following text: “Primary studies suggest that misinterpretation of wearable-assessed data may result in increased health anxiety for individuals with a chronic condition, among other possible untoward psychological and behavioral effects (84,85). For patients with PASC and post-acute viral syndromes specifically, effective symptom monitoring is a high priority. A recent systematic review of interoception in individuals with a chronic condition found that high interoceptive sensibility (an individual’s belief in their ability to detect and regulate interoceptive signals) is associated with lower symptom severity, but that interoceptive accuracy is generally lower for this population (86). Wearables may therefore be regarded by patients as crucial tools for supporting improved interoceptive sensibility, especially when symptomatic or following exertional activity. Thus, individuals who have a chronic condition are especially vulnerable to harms such as health anxiety and symptom perseveration that can result from inaccurate data or inaccurate contextualization of data. Compounding this problem, many of the core symptoms (e.g., fatigue) that patients with post-acute infection syndromes track are multifaceted and not directly reducible to wearable-assessed biometrics. To facilitate comprehensive AE assessment, formative research is needed to identify AEs that may be eventuated by consumer health wearables. Doing so would help to identify risk factors for specific AEs, as well as modifications that can attenuate risk of harms while maximizing the benefits of using these devices.” (p. 34)*

and (5) address data privacy, security, and broader digital health infrastructure issues (e.g., data transmission, IoMT integration), noting any absence of such discussion in the included reviews and citing relevant literature where appropriate.

*-Reply: We have addressed this in two places. (1) Our expanded discussion of acceptability now contains the following text (p. 33): “Potential barriers to provider acceptability include increased workload associated with reviewing additional streams of patient health data, challenges integrating wearable-assessed data into EHR systems, and concerns related to the privacy and security of these health data. Novel applications of blockchain technology may be useful both to the eventual integration of wearable-assessed data into EHR systems, and to ensuring the secure protection of these data (Almotairi et al., 2024).” (2) We have added the following to the limitations section of the manuscript (pp. 38-39): “This review also did not cover algorithms applied to biometric assessments (e.g., differences among algorithms used to estimate HRV) or data transmission, which are other key aspects of these technologies that bear upon accuracy and usability. Although we extracted reported data about acceptability broadly, we did not specifically target data privacy or security in this review. Given the role of these concerns in wearable use, this is another important limitation of this review.”*

Please submit a revised version that addresses these points, along with a detailed point-by-point response to the reviewers’ comments. We believe these revisions will substantially strengthen the manuscript and position it as a key reference in the field. We look forward to receiving your revision. [Note: HTML markup is below. Please do not edit.]

*-Reply: We concur that the revisions suggested by the editor and the reviewers have substantially strengthened our article. We appreciate the care and time taken to provide us with highly constructive feedback.*

**Reviewers' Comments:**

**Reviewer #1:**

1. The manuscript employs Smart’s model, which is effective; however, the analysis of provider acceptability is weak. While the authors mention a lack of data, they should explore why providers might not accept the technology, such as information overload or difficulty integrating it into Electronic Health Records (EHR).

*-Reply: To address this, we have added the following new text to the discussion of the manuscript (p.33): “Other gaps in the literature included limited data about acceptability from the perspective of providers, which may represent a significant barrier to implementation. Health information technology-related stress is a well-established predictor of physician burnout (75,76). Although this body of research has primarily focused on electronic health record (EHR) systems rather than adjunctive technologies such as wearables, it foreshadows broader implementation challenges that warrant targeted research. Potential barriers to provider acceptability include increased workload associated with reviewing additional streams of patient health data, challenges integrating wearable-assessed data into EHR systems, and concerns related to the privacy and security of these health data.”*

2. The manuscript notes that there is insufficient reporting on adverse events. The Discussion section should explain potential risks for individuals with Post-Acute Sequelae of SARS-CoV-2 infection (PASC), such as sleep disturbances and anxiety from false positives, even if these were not highlighted in the original reviews.

- Patient misinterpretation/incorrect contextualization of biometric data leads to increases in patient health anxiety and symptom perseveration. IMO, this is the greatest risk of using these devices with this specific patient population. Many of these patients are desperate for answers, incredibly attune to their bodies, and track symptoms very carefully. Education and data contextualization are key.
- Basic consumer health wearables not designed for effective symptom tracking within clinically unhealthy populations --> some of the core metrics that would be helpful to track for PASC pts (e.g. energy levels) are unable to be meaningfully and accurately tracked just using these devices

*-Reply: We thank the reviewer for raising this important point and offering excellent suggestions for how to address it. We have added the following text to the manuscript (pp. 34), which integrates the reviewer’s points with recent data about interoception among individuals who have a chronic illness: “Primary studies suggest that misinterpretation of wearable-assessed data may result in increased health anxiety for individuals with a chronic condition, among other possible untoward psychological and behavioral effects (84,85). For patients with PASC and post-acute viral syndromes specifically, effective symptom monitoring is a high priority. A recent systematic review of interoception in individuals with a chronic condition found that high interoceptive sensibility (an individual’s belief in their ability to detect and regulate interoceptive signals) is associated with lower symptom severity, but that interoceptive accuracy is generally lower for this population (86). Wearables may therefore be regarded by patients as crucial tools for supporting improved interoceptive sensibility, especially when symptomatic or following exertional activity. Thus, individuals who have a chronic condition are especially vulnerable to harms such as health anxiety and symptom perseveration that can result from inaccurate data or inaccurate contextualization of data. Compounding this problem, many of the core symptoms (e.g., fatigue) that patients with post-acute infection syndromes track are multifaceted and not directly reducible to wearable-assessed biometrics. To facilitate comprehensive AE assessment, formative research is needed to identify AEs that may be eventuated by consumer health wearables. Doing so would help to identify risk factors for specific AEs, as well as modifications that can attenuate risk of harms while maximizing the benefits of using these devices.”*

3. It is suggested to mention from the paper: “Personal data protection model in IoMT-blockchain on secured bit-count transmutation data encryption approach “. This paper discusses the Internet of Medical Things (IoMT) and explores methods for protecting personal medical information. Since the review examines wearable devices for outpatient monitoring, data protection is crucial to both "Acceptability" and "Clinical Utility."

*-Reply: We are grateful to have been oriented to this interesting and valuable paper. Although personal data protection models are beyond the scope of our review, we agree that the topic is relevant to our discussion and have incorporated the citation as suggested. Please see the newly added text (p. 33): “Potential barriers to provider acceptability include increased workload associated with reviewing additional streams of patient health data, challenges integrating wearable-assessed data into EHR systems, and concerns related to the privacy and security of these health data. Novel applications of blockchain technology may be useful both to the eventual integration of wearable-assessed data into EHR systems, and to ensuring the secure protection of these data (Almotairi et al., 2024).*

4. It is crucial to recognize the technological support behind these devices. The effectiveness of a wearable is not just about how accurately it measures data, but also about how well it transmits data and the algorithms used.

*-Reply: We agree with the reviewer that these are important components of wearable technologies that bear upon their accuracy and usability. Algorithms used by devices and the technical specifications of data transmission were both beyond the scope of what we extracted for this review, so we unfortunately are not able to address this in our results. In recognition of the importance of these features, we have added the following text to the Limitations subsection of our discussion (p. 38): “This review also did not cover algorithms applied to biometric assessments (e.g., differences among algorithms used to estimate HRV) or data transmission, which are other key aspects of these technologies that bear upon accuracy and usability.”*

5. The authors need to clarify whether any of the reviews discussed data privacy or security as obstacles to clinical utility. If this is not the case, it should be noted as a finding of missing evidence.

*-Reply: Although we extracted reported information about acceptability broadly, we did not specifically target data privacy or security concerns in the extraction procedures for this umbrella review. Accordingly, we have also now noted this as a limitation as follows (p. 39): “Although we extracted reported data about acceptability broadly, we did not specifically target data privacy or security in this review. Given the role of these concerns in wearable use, this is another important limitation of this review.”*

6. Expand the section on "Adverse Events" to discuss why this gap exists. Are current studies too brief to notice issues like anxiety or compulsive checking? Is the observational design of the studies to blame?

*-Reply: We concur that this is an important finding worth expanding on. We have added the following new text to the manuscript (pp. 33-34): “This review was unable to identify any review-level reporting of adverse events or adverse effects at all, including an absence of evidence for these effects. Several constraints hamper the assessment of adverse effects in consumer wearables research. Adverse effects assessment in studies of wearable technologies has never been systematized, and no articles in this review describe any formal method for AE assessment. This suggests that, as in other research areas (78,79), studies typically rely on passive monitoring (i.e., adverse events are only recorded if a participant volunteers one), rather than specific solicitation. And yet, passive monitoring under-counts AEs by up to a factor of 20 (80) compared to AE solicitation. For FDA-regulated research, which includes the investigation of medical devices, current AE assessment frameworks rely on medical and pharmacological lexica such as the Medical Dictionary for Regulatory Activities (MEDdra; 83) or Common Terminology Criteria for Adverse Events (CTCAE; 84). However, reliance on such criteria are known to systematically under-count AEs in behavioral and lifestyle interventions (83), suggesting that these lexica may not be sufficient for research on consumer wearables.”*

7. To address the revision of the discussion on Data Privacy, Security, and the Internet of Medical Things (IoMT), the following references need to be integrated into the manuscript. These references provide the essential framework for safely adopting technology.

*-Reply: Unfortunately, the references mentioned by the reviewer here were not appended to the version of the review that we received. We did incorporate the Almotairi et al., 2024 citation about IOMT-Blockchain as described above. If there were other references intended by the reviewer, we would be happy to consider those if they could be re-shared with us.*

**Reviewer #2:**

1. Strengthen clinical relevance by translating accuracy and validity findings into clear guidance for self-monitoring, screening, and clinician-supported use.

*-Reply: To address this, we have considerably revised the final subsection of the Discussion (“Are we there yet? Conclusions and recommendations about consumer wearables for outpatient health monitoring” found on pp. 40-42) to focus on clinical translation and recommendations. As part of this, we have introduced a new figure (Fig 8) that provides 5 specific recommendations for patients who are already using a wearable or want to use one, guided by findings from our review.*

2. Clarify applicability to PASC and post-infection syndromes, explicitly distinguishing direct evidence from extrapolated findings.

*-Reply: We appreciate the opportunity clarify this point. Given that PASC only emerged as a recognized clinical diagnosis in 2020 and post-acute viral syndromes are under-researched, we intentionally applied broad search and inclusion criteria for this review (due to the paucity of review-level evidence for PASC and post-infection syndromes specifically). We previously noted in the Discussion that only 7 of 42 reviews targeted populations with a clinical or morbidity characteristic at all. To address this feedback, we have added the following expanded discussion to our limitations (p. 39): “As noted previously, this umbrella review employed broad search and inclusion criteria given that PASC only emerged as a recognized diagnosis in 2020 and limited data for individuals with others post-acute viral syndromes exist. Reflecting this paucity of data and the broader tendency for wearable research to focus on healthy populations, only 7 of 42 reviews examined populations with a clinical or morbidity characteristics at all. Accordingly, this review provides extrapolated findings with relevance to PASC and post-acute infection syndromes and should not be interpreted as direct evidence.”*

3. Improve synthesis of heterogeneous evidence using clearer summaries, visuals, and identification of consistent patterns across devices and contexts

*-Reply: We thought this was an excellent suggestion and carefully weighed how to best provide a concise visual aid that highlights consistent patterns without inadvertently masking the heterogeneity of the evidence. To accomplish this, we have created a new figure (Fig 4 of this revision) that provides a heat map of (1) the volume of evidence for each biometric, and (2) the overall evaluation of accuracy based on that evidence, alongside (3) a brief summary with any major cautions noted. We have also introduced Fig 8, which provides summative recommendations for patients who are already using a wearable or want to use one.*

4. Address equity and bias more explicitly, emphasizing demographic gaps, differential accuracy, and implications for safety and access.

*-Reply: To address this, we have expanded a subsection of the Discussion (now titled: “Equity and underrepresentation: Do wearables work equally well for everyone?”) To this section, we have added the following new text (pp. 35-36): “This review identified a concerning lack of review-level evaluation of device performance across diverse demographic groups. While age (reported in 74.5% of reviews) and gender (reported in 69% of reviews) were frequently addressed, race and ethnicity were rarely examined, appearing in only 7% (k=3) reviews and fewer than 5% (k=2) reviews, respectively. When demographic factors were examined, findings often suggested diminished performance for some biometrics and devices based on these characteristics—for example, the finding that photoplethysmography (PPG) sensors (used commonly for HR measurement in wristwatch devices) may be less accurate in individuals with darker skin tones (44). Socioeconomic status was also infrequently included (14% or k=6 reviews), despite its clear relevance to wearable device access. Many wearable devices depend on smartphone pairing and reliable internet connectivity, which may systematically exclude individuals with lower incomes or those living in rural areas. These equity issues are highly intersectional, given that individuals from lower income backgrounds are disproportionately represented among historically underrepresented racial and ethnic groups. Together, these gaps raise concerns about the generalizability and equity of the current evidence, and highlight a need for more inclusive primary studies and consistent reporting of demographic characteristics in future research.”*
