# Supplementary material for: Can consumer wearables support outpatient health monitoring for patients with post-acute infection syndromes? A systematic umbrella review of accuracy, validity, and clinical utility data
Source: PLOS Digit Health. 2026 Jun 8;5(6):e0001124. doi: 10.1371/journal.pdig.0001124 (PMC13245765; doi:10.1371/journal.pdig.0001124)
Supplement: Peer review history

# Peer review: Decision Letter 0

**DOI:** 10.1371/journal.pdig.0001124.r001

**Authors:** Hyunjung Kwak, Xiangbin Meng

4 Feb 2026

PDIG-D-25-01036

Can consumer wearables support outpatient health monitoring for patients with post-acute infection syndromes? A systematic umbrella review of accuracy, validity, and clinical utility data

PLOS Digital Health

Dear Dr. Kaplan,

Thank you for submitting your manuscript to PLOS Digital Health. After careful consideration, we feel that it has merit but does not fully meet PLOS Digital Health's publication criteria as it currently stands. Therefore, we invite you to submit a revised version of the manuscript that addresses the points raised during the review process.

Please submit your revised manuscript by Apr 05 2026 11:59PM. If you will need more time than this to complete your revisions, please reply to this message or contact the journal office at digitalhealth@plos.org. When you're ready to submit your revision, log on to https://www.editorialmanager.com/pdig/ and select the 'Submissions Needing Revision' folder to locate your manuscript file.

Please include the following items when submitting your revised manuscript:

* A letter that responds to each point raised by the editor and reviewer(s). You should upload this letter as a separate file labeled 'Response to Reviewers'. This file does not need to include responses to any formatting updates and technical items listed in the 'Journal Requirements' section below.

* A marked-up copy of your manuscript that highlights changes made to the original version. You should upload this as a separate file labeled 'Revised Manuscript with Track Changes'.

* An unmarked version of your revised paper without tracked changes. You should upload this as a separate file labeled 'Manuscript'.

If you would like to make changes to your financial disclosure, competing interests statement, or data availability statement, please make these updates within the submission form at the time of resubmission. Guidelines for resubmitting your figure files are available below the reviewer comments at the end of this letter.

We look forward to receiving your revised manuscript.

Kind regards,

Xiangbin Meng

Academic Editor

PLOS Digital Health

Hyunjung Kwak

Section Editor

PLOS Digital Health

Leo Anthony Celi

Editor-in-Chief

PLOS Digital Health

orcid.org/0000-0001-6712-6626

**Journal Requirements:**

1.  Please amend your detailed Financial Disclosure statement. This is published with the article. It must therefore be completed in full sentences and contain the exact wording you wish to be published.

i. Please clarify all sources of financial support for your study. List the grants, grant numbers, and organizations that funded your study, including funding received from your institution. Please note that suppliers of material support, including research materials, should be recognized in the Acknowledgements section rather than in the Financial Disclosure.

ii. State the initials, alongside each funding source, of each author to receive each grant. For example: "This work was supported by the National Institutes of Health (####### to AM; ###### to CJ) and the National Science Foundation (###### to AM)."

iii. State what role the funders took in the study. If the funders had no role in your study, please state: “The funders had no role in study design, data collection and analysis, decision to publish, or preparation of the manuscript.”

iv. If any authors received a salary from any of your funders, please state which authors and which funders.

2. Please send a completed 'Competing Interests' statement, including any COIs declared by your co-authors. If you have no competing interests to declare, please state "The authors have declared that no competing interests exist". Otherwise please declare all competing interests beginning with the statement "I have read the journal's policy and the authors of this manuscript have the following competing interests:"

3. Please upload separate figure files in .tif or .eps format. Also, remove the figures from your manuscript file but keep the legends.

For more information about figure files please see our guidelines:

https://journals.plos.org/digitalhealth/s/figures

https://journals.plos.org/digitalhealth/s/figures#loc-file-requirements

4. We have noticed that you have uploaded Supporting Information files, but you have not included a list of legends. Please add a full list of legends for your Supporting Information files after the references list.

5. As required by our policy on Data Availability, please ensure your manuscript or supplementary information includes the following:

A numbered table of all studies identified in the literature search, including those that were excluded from the analyses.

For every excluded study, the table should list the reason(s) for exclusion.

If any of the included studies are unpublished, include a link (URL) to the primary source or detailed information about how the content can be accessed.

A table of all data extracted from the primary research sources for the systematic review and/or meta-analysis. The table must include the following information for each study:

Name of data extractors and date of data extraction

Confirmation that the study was eligible to be included in the review.

All data extracted from each study for the reported systematic review and/or meta-analysis that would be needed to replicate your analyses.

If data or supporting information were obtained from another source (e.g. correspondence with the author of the original research article), please provide the source of data and dates on which the data/information were obtained by your research group.

If applicable for your analysis, a table showing the completed risk of bias and quality/certainty assessments for each study or outcome.  Please ensure this is provided for each domain or parameter assessed. For example, if you used the Cochrane risk-of-bias tool for randomized trials, provide answers to each of the signalling questions for each study. If you used GRADE to assess certainty of evidence, provide judgements about each of the quality of evidence factor. This should be provided for each outcome.

An explanation of how missing data were handled.

This information can be included in the main text, supplementary information, or relevant data repository. Please note that providing these underlying data is a requirement for publication in this journal, and if these data are not provided your manuscript might be rejected.

If the reviewer comments include a recommendation to cite specific previously published works, please review and evaluate these publications to determine whether they are relevant and should be cited. There is no requirement to cite these works unless the editor has indicated otherwise.

**Additional Editor Comments (if provided):**

Dear Dr. Kaplan and co-authors,

We thank you for submitting your manuscript to *PLOS Digital Health* and for your thorough and timely work on this highly relevant topic. This pre-registered systematic umbrella review provides a valuable synthesis of evidence on the accuracy, validity, and clinical utility of consumer wearables for biometrics relevant to post-acute infection syndromes, including Long COVID (PASC). The rigorous methodology—adherence to PRISMA guidelines, PROSPERO registration, AMSTAR 2.0 quality appraisal, double data extraction, and full data availability on OSF—is exemplary. The inclusion of 42 reviews covering more than 30 brands and over 150 device series offers comprehensive coverage, and the identification of stronger evidence for heart rate measurement and atrial fibrillation detection, alongside limited support for other biometrics, is clearly presented. The supplementary device-level benchmarking tables and especially the innovative living review repository on the Open Science Framework are significant strengths that enhance the practical utility and longevity of this work for researchers, clinicians, and patients.

Both reviewers found the manuscript technically sound, well-written, and important, but identified opportunities to strengthen its impact. To better serve our interdisciplinary readership, we ask you to revise the manuscript to (1) provide clearer clinical translation of the findings into practical guidance for self-monitoring, activity pacing, screening, and clinician-supported use in PASC and related syndromes, explicitly distinguishing direct evidence from PASC populations versus extrapolation from other groups; (2) improve synthesis and presentation of the heterogeneous evidence through additional summaries, tables, or visual aids that highlight consistent patterns and facilitate interpretation; (3) expand discussion of equity considerations, including demographic gaps in validation studies (e.g., skin tone, race/ethnicity, age, socioeconomic status), differential accuracy, and implications for safety and access; (4) deepen analysis of provider acceptability (e.g., EHR integration challenges, information overload, algorithmic transparency), potential adverse events or harms in PASC patients (e.g., anxiety from false alerts, compulsive checking), and reasons for gaps in reporting these outcomes; and (5) address data privacy, security, and broader digital health infrastructure issues (e.g., data transmission, IoMT integration), noting any absence of such discussion in the included reviews and citing relevant literature where appropriate.

Please submit a revised version that addresses these points, along with a detailed point-by-point response to the reviewers’ comments. We believe these revisions will substantially strengthen the manuscript and position it as a key reference in the field. We look forward to receiving your revision.

[Note: HTML markup is below. Please do not edit.]

**Reviewers' Comments:**

Reviewer's Responses to Questions

**Comments to the Author**

1. Does this manuscript meet PLOS Digital Health’s publication criteria? Is the manuscript technically sound, and do the data support the conclusions? The manuscript must describe methodologically and ethically rigorous research with conclusions that are appropriately drawn based on the data presented.

Reviewer #1: Yes

Reviewer #2: Yes

**********

2. Has the statistical analysis been performed appropriately and rigorously?

Reviewer #1: Yes

Reviewer #2: Yes

**********

3. Have the authors made all data underlying the findings in their manuscript fully available (please refer to the Data Availability Statement at the start of the manuscript PDF file)?

The PLOS Data policy requires authors to make all data underlying the findings described in their manuscript fully available without restriction, with rare exception. The data should be provided as part of the manuscript or its supporting information, or deposited to a public repository. For example, in addition to summary statistics, the data points behind means, medians and variance measures should be available. If there are restrictions on publicly sharing data—e.g. participant privacy or use of data from a third party—those must be specified.

Reviewer #1: Yes

Reviewer #2: Yes

**********

4. Is the manuscript presented in an intelligible fashion and written in standard English?

PLOS Digital Health does not copyedit accepted manuscripts, so the language in submitted articles must be clear, correct, and unambiguous. Any typographical or grammatical errors should be corrected at revision, so please note any specific errors here.

Reviewer #1: Yes

Reviewer #2: Yes

**********

5. Review Comments to the Author

Please use the space provided to explain your answers to the questions above. You may also include additional comments for the author, including concerns about dual publication, research ethics, or publication ethics. (Please upload your review as an attachment if it exceeds 20,000 characters)

Reviewer #1: 1. The manuscript employs Smart’s model, which is effective; however, the analysis of provider acceptability is weak. While the authors mention a lack of data, they should explore why providers might not accept the technology, such as information overload or difficulty integrating it into Electronic Health Records (EHR).

2. The manuscript notes that there is insufficient reporting on adverse events. The Discussion section should explain potential risks for individuals with Post-Acute Sequelae of SARS-CoV-2 infection (PASC), such as sleep disturbances and anxiety from false positives, even if these were not highlighted in the original reviews.

3. It is suggested to mention from the paper: “Personal data protection model in IoMT-blockchain on secured bit-count transmutation data encryption approach “. This paper discusses the Internet of Medical Things (IoMT) and explores methods for protecting personal medical information. Since the review examines wearable devices for outpatient monitoring, data protection is crucial to both "Acceptability" and "Clinical Utility."

4. It is crucial to recognize the technological support behind these devices. The effectiveness of a wearable is not just about how accurately it measures data, but also about how well it transmits data and the algorithms used.

5. The authors need to clarify whether any of the reviews discussed data privacy or security as obstacles to clinical utility. If this is not the case, it should be noted as a finding of missing evidence.

6. Expand the section on "Adverse Events" to discuss why this gap exists. Are current studies too brief to notice issues like anxiety or compulsive checking? Is the observational design of the studies to blame?

7. To address the revision of the discussion on Data Privacy, Security, and the Internet of Medical Things (IoMT), the following references need to be integrated into the manuscript. These references provide the essential framework for safely adopting technology.

Reviewer #2: 1. Strengthen clinical relevance by translating accuracy and validity findings into clear guidance for self-monitoring, screening, and clinician-supported use.

2. Clarify applicability to PASC and post-infection syndromes, explicitly distinguishing direct evidence from extrapolated findings.

3. Improve synthesis of heterogeneous evidence using clearer summaries, visuals, and identification of consistent patterns across devices and contexts.

4. Address equity and bias more explicitly, emphasizing demographic gaps, differential accuracy, and implications for safety and access.

**********

6. PLOS authors have the option to publish the peer review history of their article (what does this mean?). If published, this will include your full peer review and any attached files.

**Do you want your identity to be public for this peer review?** If you choose “no”, your identity will remain anonymous but your review may still be made public.

For information about this choice, including consent withdrawal, please see our Privacy Policy.

Reviewer #1: No

Reviewer #2: No

**********

[NOTE: If reviewer comments were submitted as an attachment file, they will be attached to this email and accessible via the submission site. Please log into your account, locate the manuscript record, and check for the action link "View Attachments". If this link does not appear, there are no attachment files.]

**Figure resubmission:**

While revising your submission, we strongly recommend that you use PLOS’s NAAS tool (https://ngplosjournals.pagemajik.ai/artanalysis) to test your figure files. NAAS can convert your figure files to the TIFF file type and meet basic requirements (such as print size, resolution), or provide you with a report on issues that do not meet our requirements and that NAAS cannot fix.

After uploading your figures to PLOS’s NAAS tool - https://ngplosjournals.pagemajik.ai/artanalysis, NAAS will process the files provided and display the results in the "Uploaded Files" section of the page as the processing is complete. If the uploaded figures meet our requirements (or NAAS is able to fix the files to meet our requirements), the figure will be marked as "fixed" above. If NAAS is unable to fix the files, a red "failed" label will appear above. When NAAS has confirmed that the figure files meet our requirements, please download the file via the download option, and include these NAAS processed figure files when submitting your revised manuscript.

**Reproducibility:**

To enhance the reproducibility of your results, we recommend that authors of applicable studies deposit laboratory protocols in protocols.io, where a protocol can be assigned its own identifier (DOI) such that it can be cited independently in the future. Additionally, PLOS ONE offers an option to publish peer-reviewed clinical study protocols. Read more information on sharing protocols at https://plos.org/protocols?utm_medium=editorial-email&utm_source=authorletters&utm_campaign=protocols

# Author comment: Author response to Decision Letter 1

**DOI:** 10.1371/journal.pdig.0001124.r002

3 Apr 2026

Attachment Submitted filename: PASC Review Response to Reviewers.docx

# Peer review: Decision Letter 1

**DOI:** 10.1371/journal.pdig.0001124.r003

**Authors:** Po-Chih Kuo, Xiangbin Meng

5 May 2026

Can consumer wearables support outpatient health monitoring for patients with post-acute infection syndromes? A systematic umbrella review of accuracy, validity, and clinical utility data

PDIG-D-25-01036R1

Dear Kaplan,

We are pleased to inform you that your manuscript 'Can consumer wearables support outpatient health monitoring for patients with post-acute infection syndromes? A systematic umbrella review of accuracy, validity, and clinical utility data' has been provisionally accepted for publication in PLOS Digital Health.

Before your manuscript can be formally accepted you will need to complete some formatting changes, which you will receive in a follow-up email from a member of our team.

Please note that your manuscript will not be scheduled for publication until you have made the required changes, so a swift response is appreciated.

IMPORTANT: The editorial review process is now complete. PLOS will only permit corrections to spelling, formatting or significant scientific errors from this point onwards. Requests for major changes, or any which affect the scientific understanding of your work, will cause delays to the publication date of your manuscript.

If your institution or institutions have a press office, please notify them about your upcoming paper to help maximize its impact. If they'll be preparing press materials, please inform our press team as soon as possible -- no later than 48 hours after receiving the formal acceptance. Your manuscript will remain under strict press embargo until 2 pm Eastern Time on the date of publication. For more information, please contact digitalhealth@plos.org.

Thank you again for supporting Open Access publishing; we are looking forward to publishing your work in PLOS Digital Health.

Best regards,

Xiangbin Meng, M.D., Ph.D.

Academic Editor

PLOS Digital Health

***********************************************************

**Additional Editor Comments (if provided):**

Thank you for the thoughtful and comprehensive revision of your manuscript. The revised version has addressed the key concerns raised by the reviewers and editor, and the manuscript has been substantially strengthened as a result. The additions regarding clinical applicability, evidence synthesis, equity, implementation challenges, and safety considerations are particularly valuable. I am pleased to inform you that the manuscript is now suitable for acceptance.

**Reviewer Comments (if any, and for reference):**

Reviewer's Responses to Questions

**Comments to the Author**

1. If the authors have adequately addressed your comments raised in a previous round of review and you feel that this manuscript is now acceptable for publication, you may indicate that here to bypass the “Comments to the Author” section, enter your conflict of interest statement in the “Confidential to Editor” section, and submit your "Accept" recommendation.

Reviewer #1: All comments have been addressed

Reviewer #2: All comments have been addressed

**********

2. Does this manuscript meet PLOS Digital Health’s publication criteria? Is the manuscript technically sound, and do the data support the conclusions? The manuscript must describe methodologically and ethically rigorous research with conclusions that are appropriately drawn based on the data presented.

Reviewer #1: Yes

Reviewer #2: Yes

**********

3. Has the statistical analysis been performed appropriately and rigorously?

Reviewer #1: Yes

Reviewer #2: Yes

**********

4. Have the authors made all data underlying the findings in their manuscript fully available (please refer to the Data Availability Statement at the start of the manuscript PDF file)?

The PLOS Data policy requires authors to make all data underlying the findings described in their manuscript fully available without restriction, with rare exception. The data should be provided as part of the manuscript or its supporting information, or deposited to a public repository. For example, in addition to summary statistics, the data points behind means, medians and variance measures should be available. If there are restrictions on publicly sharing data—e.g. participant privacy or use of data from a third party—those must be specified.

Reviewer #1: Yes

Reviewer #2: Yes

**********

5. Is the manuscript presented in an intelligible fashion and written in standard English?

PLOS Digital Health does not copyedit accepted manuscripts, so the language in submitted articles must be clear, correct, and unambiguous. Any typographical or grammatical errors should be corrected at revision, so please note any specific errors here.

Reviewer #1: Yes

Reviewer #2: (No Response)

**********

6. Review Comments to the Author

Please use the space provided to explain your answers to the questions above. You may also include additional comments for the author, including concerns about dual publication, research ethics, or publication ethics. (Please upload your review as an attachment if it exceeds 20,000 characters)

Reviewer #1: The authors have addressed all reviewer comments and methodological concerns from the previous round. The revised version meets the high standards of this journal and is now suitable for acceptance.

1. The authors addressed all concerns from the initial review, showing their commitment to improvement.

2. The manuscript's structure has improved, especially in the Discussion section, enhancing readability and flow.

3. The methods are solid, with additional details on data extraction ensuring transparency and accuracy.

4. This systematic umbrella review offers an important, original contribution to digital health, providing a clear overview of wearable accuracy for post-acute infection syndromes.

5. Validation is stronger thanks to visual aids like a heatmap and clinical recommendation figures, which improve data interpretation.

6. The overall presentation quality has increased, with more focus on equity and previously overlooked issues.

Reviewer #2: (No Response)

**********

7. PLOS authors have the option to publish the peer review history of their article (what does this mean?). If published, this will include your full peer review and any attached files.

**Do you want your identity to be public for this peer review?** If you choose “no”, your identity will remain anonymous but your review may still be made public.

For information about this choice, including consent withdrawal, please see our Privacy Policy.

Reviewer #1: No

Reviewer #2: No

**********
